# Single-cell sequencing reveals suppressive transcriptional programs regulated by MIS/AMH in neonatal ovaries

**DOI:** 10.1073/pnas.2100920118

**Published:** 2021-05-12

**Authors:** Marie-Charlotte Meinsohn, Hatice D. Saatcioglu, Lina Wei, Yi Li, Heiko Horn, Maeva Chauvin, Motohiro Kano, Ngoc Minh Phuong Nguyen, Nicholas Nagykery, Aki Kashiwagi, Wesley R. Samore, Dan Wang, Esther Oliva, Guangping Gao, Mary E. Morris, Patricia K. Donahoe, David Pépin

**Affiliations:** ^a^Pediatric Surgical Research Laboratories, Massachusetts General Hospital, Boston, MA 02114;; ^b^Department of Surgery, Harvard Medical School, Boston, MA 02115;; ^c^Stanley Center, Broad Institute of MIT and Harvard, Cambridge, MA 02142;; ^d^Department of Pathology, Massachusetts General Hospital, Boston, MA 02114;; ^e^Department of Pathology, Harvard Medical School, Boston MA 02115;; ^f^Horae Gene Therapy Center, University of Massachusetts, Worcester, MA 01605;; ^g^Department of Gynecology, Massachusetts General Hospital, Boston, MA 02114;; ^h^Department of Obstetrics and Gynecology, Harvard Medical School, Boston MA 02115

**Keywords:** Müllerian inhibiting substance, ovarian reserve, ovary, granulosa cells, scRNA-seq

## Abstract

Müllerian inhibiting substance (MIS/AMH), produced by granulosa cells of growing follicles, is an important regulator of folliculogenesis and follicle development. Treatment with exogenous MIS in mice suppresses follicle development and prevents ovulation. To investigate the mechanisms by which MIS inhibits follicle development, we performed single-cell RNA sequencing of whole neonatal ovaries treated with MIS at birth and analyzed at postnatal day 6, coinciding with the first wave of follicle growth. We identified distinct transcriptional signatures associated with MIS responses in the ovarian cell types. MIS treatment inhibited proliferation in granulosa, surface epithelial, and stromal cell types of the ovary and elicited a unique signature of quiescence in granulosa cells. In addition to decreasing the number of growing preantral follicles, we found that MIS treatment uncoupled the maturation of germ cells and granulosa cells. In conclusion, MIS suppressed neonatal follicle development by inhibiting proliferation, imposing a quiescent cell state, and preventing granulosa cell differentiation.

## Significance

This study improves our understanding of the role of Müllerian inhibiting substance (MIS/AMH) in antagonizing primordial follicle activation. By applying scRNA-seq methods to neonatal mouse ovaries, we revealed transcriptional signatures associated with MIS treatment in ovarian cell types present during the first wave of folliculogenesis and follicle development. We showed that MIS inhibits pregranulosa cell differentiation and the proliferation of ovarian surface epithelium and stromal cells. MIS also uncoupled granulosa and germ cell maturation, leading to abnormal development of activated follicles. These findings identify markers and pathways related to primordial follicle quiescence that could be targeted in contraception, preservation of ovarian reserve during aging or chemotherapy, or synchronization of preantral follicle growth for IVF.

The ovarian reserve, the total number of eggs present in the ovary at any given time, peaks before birth in humans and rodents and immediately declines through fetal oocyte attrition (1). Individual follicles are formed as a result of the breakdown of germ cell-containing cysts, and the invasion of somatic pregranulosa cells that envelop individual oocytes to form a single layer of flattened granulosa cells that characterize the primordial follicle. The timing of this process differs among mammals, occurring during fetal development in humans, at around 18 wk postfertilization (2), or immediately after birth in rodents (3), between postnatal day 1 (PND1) and PND3. While most of the primordial follicles are described as “dormant” or “resting,” the initial period immediately following their formation is marked by a wave of both atresia and activation, which is dependent on the location of the follicle (4), the former more medial, the latter more peripheral. As activated primordial follicles transition irreversibly to primary follicles, granulosa cells differentiate into a cuboidal phenotype, and the oocytes concomitantly differentiate and grow (5, 6). During preantral follicle maturation, granulosa cells continue to proliferate, forming multiple layers, commensurate with the growth of the oocyte, which defines the secondary follicle. Thereafter, tertiary or antral follicles form an antral cavity marked by further differentiation of granulosa cells into mural and cumulus layers. During follicle development, thecal layers also form from adjacent somatic cells to support the follicle with both structural and steroidogenic functions. While most activated follicles succumb to atresia, in a small subset, the process ultimately culminates, after puberty, with the release or ovulation of an oocyte, a process that continues iteratively until menopause (7).

The mechanisms that trigger or inhibit the activation of primordial follicles and regulate early preantral follicle growth remain largely unknown. Müllerian inhibiting substance (MIS), also known as anti-Müllerian hormone (AMH), is known for its role in male sex differentiation (8). Its serum levels have also been used as a surrogate measure of ovarian reserve by enzyme-linked immunosorbent assays (ELISAs) of blood (9). Indeed, MIS plays an important role in the differentiation of the urogenital ridge (8), leading to the regression of the Müllerian ducts and the development of testes in males, while in females it is thought to be a key hormone in the development of the uterus and ovary prenatally and postnatally (10–12).

Transgenic female mice overexpressing MIS ectopically during embryo development develop gonadal dysgenesis (13), supporting the hypothesis that MIS plays an inhibitory role in early follicle assembly. This has been corroborated by in vitro experiments demonstrating inhibition of cyst breakdown in MIS-treated newborn rat ovaries (3). Postnatally, in females, MIS is mainly produced by granulosa cells of secondary and early antral follicles and acts through its specific receptor, MISR2 (AMHR2), which is expressed in granulosa cells of all types and in ovarian surface epithelium (OSE) (14). MIS produced by growing follicles is thought to provide a negative feedback to prevent primordial follicle activation, a function that has been supported by in vitro and in vivo studies in rodents and humans (15–18).

Culture of early human ovarian cortical sections, which include only primordial and primary follicles, display immediate recruitment or activation of nearly all primordial follicles in the absence of endogenous inhibitory signals (19). However, treatment of cultured PND4 rat ovaries with MIS significantly reduced activation and consequent transition of primordial follicles to primary follicles when compared to controls (20). Conversely, in MIS germline knockout mice, females experience an accelerated decline of ovarian reserve as they age (21), due to excessive activation of primordial follicles, although this decline is not associated with a reduction in fertility, suggesting other limiting factors in addition to MIS (21).

Here, we aimed to understand the mechanisms by which MIS inhibits the first wave of follicular activation. In this context, we administered superphysiological doses of MIS immediately after birth and performed transcriptomic analyses with single-cell RNA sequencing (scRNA-seq). We hypothesized that comparing gene expression signatures of early control and MIS-treated ovaries at PND6, when activated primordial follicles normally begin their growth (2), would reveal previously uncharacterized markers of primordial follicle quiescence and activation, and thereby help elucidate the transcriptional programs associated with ovarian suppression by MIS.

## Results

### *Misr2* Is Expressed in Granulosa Cells of Primordial Follicles and Exogenous MIS Inhibits Their Activation.

**Fig. 1.**
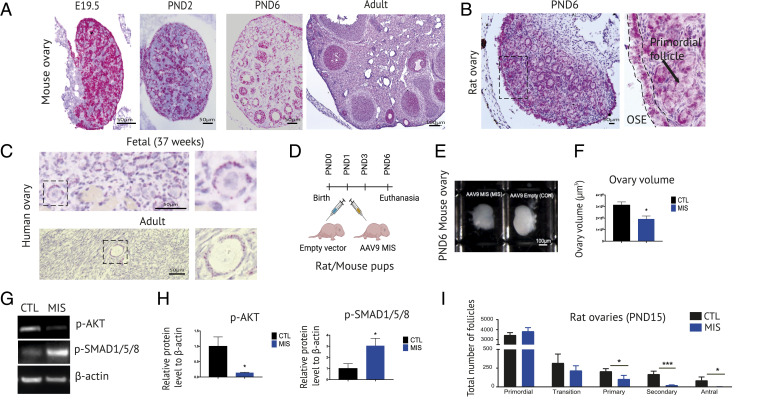
*Misr2* is expressed in primordial follicle granulosa cells and exogenous MIS inhibits their activation. (*A*) *Misr2* expression pattern by RNA in situ hybridization (RNAish) in mouse ovaries. (Scale bars, 100 μm for adult ovaries; 50 μm for all of the other sections.) (*B*) *Misr2* expression pattern by RNAish in PND6 rat ovaries. (Scale bar, 50 μm.) (*C*) *Misr2* expression pattern by RNAish in human fetal and adult ovary. (Scale bars, 50 μm.) (*D*) Illustration of the experimental design with pups receiving empty vector (CTL) or AAV9-MIS (MIS) injection on postnatal day 1 (PND1) and killed on PND6. (*E*) Gross images of CTL- or MIS-treated mice ovaries on PND6. (Scale bars, 100 μm.) (*F*) Volume of CTL- and MIS-treated PND6 mice ovaries (three ovaries from three animals/group). (*G*) p-AKT, p-SMAD 1/5/8, and β-actin levels by Western blot of CTL- and MIS-treated rat ovaries at PND6. (*H*) Quantification of p-AKT and p-SMAD1/5/8 Western blots rationalized on β-actin (mean ± SEM based on three replicates; *P < 0.05). (*I*) Quantification and classification of PND15 CTL- and MIS-treated rat ovaries based on serial sectioning and H&E staining (*n* = 3 per group). All types of follicles presenting their nucleus in the plane of section were counted in every other 5-μm section.

To determine the role of MIS on follicular development, we first confirmed the expression of its specific receptor, *Misr2*, during the developmental trajectory of the follicle. We compared spatiotemporal expression of MISR2 in mouse, rat, and human ovaries (Fig. 1 *A*–*C* and *SI Appendix*, Fig. S1). Robust levels of *Misr2* were detected in mouse ovaries at PND6 as revealed by whole-ovary RT-qPCR when compared to positive tissues such as testis or other presumed negative organs in adults (*SI Appendix*, Fig. S1*A*). To examine the cell types expressing *Misr2*, we used RNA in situ hybridization (RNAish) with the RNAscope system. In sagittal histological sections of mouse fetuses, we showed highly restricted expression in the well-established Misr2+ mesenchyme of the embryonic Müllerian duct (14) and, unexpectedly, in the muscle of the tongue, at embryonic day 17.5 (E17.5) (*SI Appendix*, Fig. S1*B*). In the neonatal male, *Misr2* expression was prominent in Sertoli cells of the testis, and it was shown to be specific by the absence of signal using the negative control probe DapB (*SI Appendix*, Fig. S1*C*). In a developmental time series in the mouse and rat, we confirmed robust *Misr2* expression throughout the development of the ovary (Fig. 1 *A* and *B*). Indeed, positive staining could be seen in the ovary from E19.5 to PND2, both in pregranulosa cells of germ cysts and OSE. Following germ-cyst breakdown, expression was maintained in OSE, and granulosa cells of primordial, primary, and early secondary follicles at PND6, a pattern that was retained in the adult with the addition of large antral follicles (*SI Appendix*, Fig. S1*D*). As expected, *Misr2* expression dramatically decreased in the corpus luteum (CL) and was low to undetectable in the theca and medullary stroma (*SI Appendix*, Fig. S1*D*). This pattern of expression was conserved in humans with robust expression of *MISR2* in granulosa cells of primordial follicles of both fetal (37 wk) (Fig. 1*C*) and adult (37-y) ovaries (Fig. 1*C*), as well as in granulosa cells of larger follicles, and, in contrast to rodents, in theca cells (*SI Appendix*, Fig. S1*E*). To evaluate the role of MIS in early follicular development, we chose to examine ovaries at PND6, an ideal timepoint when germ cell cyst breakdown is fully completed, the number of primordial follicles is high, and follicles have begun their coordinated maturation to primary and early secondary stages (22). To dissect the effect of MIS on neonatal primordial follicle activation and early follicular growth, we injected PND1 mice and rats with an adeno-associated viral vectors (AAV9) (2.5E10 particles/pup) carrying a highly bioactive human MIS transgene (Fig. 1*D*), which infects tissues and results in secretion of MIS in the circulation, as detected by ELISA (10, 23) (*SI Appendix*, Fig. S1*F*), thus acting on the ovary *in trans*. We then evaluated the effect of this continuous exposure to exogenous MIS on the ovaries at PND6, by confirming activation of the MISR2 pathway, as evidenced by the induction of the downstream canonical SMAD signaling (p-SMAD1/5/8), and down-regulation of the p-AKT activation pathway by Western blot (Fig. 1 *G* and *H*). Ovarian suppression was evident by a reduction in the size of the rat and mouse ovaries, as illustrated and quantified in PND6 mouse ovaries (Fig. 1 *E* and *F*). The ovarian suppression was further quantified by total follicle counts through serial sectioning of PND15 rat ovaries, a timepoint that allows some follicles to reach the early antral stage in controls (Fig. 1*I*). While the primordial follicle population number trended higher in the MIS-treated group, follicle counts showed a significant decrease in the number of growing follicles of every stage, from primary to antral.

### scRNA-seq of PND6 Ovaries Treated with MIS Reveals Profound Alterations of Cellular States across a Variety of Cell Types.

**Fig. 2.**
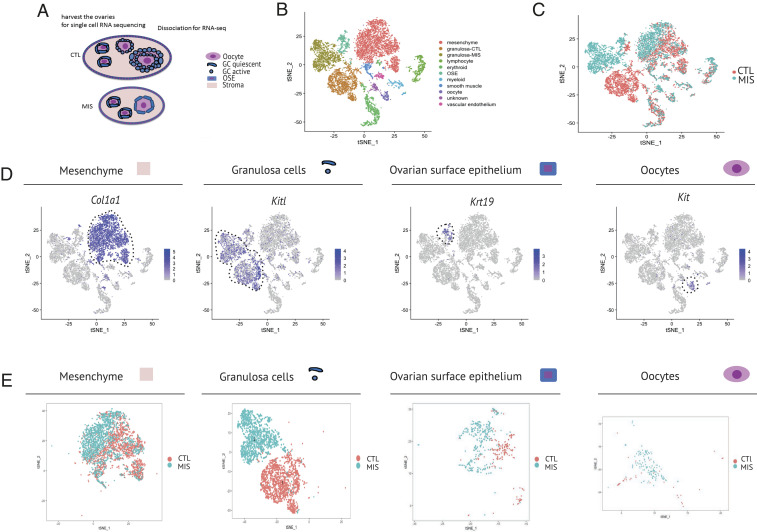
Single-cell RNA-sequencing (scRNA-seq) of neonatal ovaries reveals gene signatures of granulosa cells treated with MIS. (*A*) Schema of cell types detected in the scRNA-seq dataset of the PND6 mice ovary treated subcutaneously with empty vector (CTL) or AAV9-MIS (MIS) on PND1 (two replicates of *n* = 3). (*B*) *t*-distributed stochastic neighbor embedding (*t*-SNE) plot of unbiased clustering of 9,437 ovarian cells, where each color-coded cluster represents one cell type/state. (*C*) *t*-SNE plot of unbiased clustering of ovarian cells color-coded by treatment (CTL, orange; MIS, blue). (*D*) Gene expression levels of representative cell type-specific markers for each cluster overlaid on *t*-SNE plots. (*E*) *t*-SNE plots of different cell types (mesenchyme, granulosa, OSE, oocyte) of the ovaries dissected from CTL (orange)- and MIS (blue)-treated mice.

To understand how MIS might regulate the complex multicellular programs underlying folliculogenesis, we performed scRNA-seq in PND6 control and MIS-treated mouse ovaries, following treatment with AAV9-MIS or AAV9-empty control at PND1 (Fig. 1*D*). Ovaries of three controls (treated with 2.5E10 empty vector particles/pup on PND1) and three MIS-treated animals (treated with 2.5E10 particles of AAV9-MIS/pup on PND1) were harvested at PND6 and digested in a mixture of proteases into single-cell suspensions (Fig. 2*A*). This experiment was repeated so that two inDROP libraries were sequenced per condition, demultiplexed, normalized, and analyzed together using the Seurat v2 package in R, as previously described (Fig. 2*B* and *SI Appendix*, Fig. S2 *A* and *B*) (24). Following quality checks to exclude cells with low gene counts or high percentage of mitochondrial genes, we normalized and analyzed the combined data in Seurat. The final R object contained 9,437 cells partitioned into 18 clusters (*SI Appendix*, Fig. S2*A*). To simplify downstream analysis, some clusters were combined based on expression of specific markers into consolidated granulosa and mesenchyme superclusters, to form 11 groups: mesenchymal (3,273), granulosa-CTL (1,695), granulosa-MIS (1,451), lymphocytes (580), erythroid (905), OSE (459), myeloid (305), smooth muscle (246), oocyte (188), vascular endothelium (156), along with a low-information cluster with few cells and high mitochondrial gene counts, which was censored from further analysis (179) (*SI Appendix*, Fig. S2*C*). We assigned identity to cell types of each cluster using previously reported cell type markers enriched by adjusted *P* value (*SI Appendix*, Table S1). The top five genes for each cluster (by fold expression over average) are presented in a heatmap (*SI Appendix*, Fig. S2*D*).

While we observed a high concordance of the *t*-SNE plots of paired experimental replicates of control and MIS samples, a clear separation of distribution based on treatment can also be seen in *t*-SNE clustering (Fig. 2*C*), with some clusters, such as granulosa cells, composed almost exclusively of control or MIS-treated cells (Fig. 2*C*). The expression levels of well-characterized marker genes for the main cell types of the ovary were shown to be highly specific to their corresponding clusters, such as *Kitl* (granulosa cells), *Krt19* (OSE), *Kit* (oocyte), and *Col1a1* (mesenchyme) (Fig. 2*D*). Furthermore, while MIS treatment caused a differential clustering only in granulosa cells, clear subclustering differences based on treatment can be seen in other cell types such as OSE and mesenchymal cells, but not in oocytes (Fig. 2*E*).

### Exogenous MIS Induces Common Transcriptional Signatures in Granulosa and OSE Cells.

**Fig. 3.**
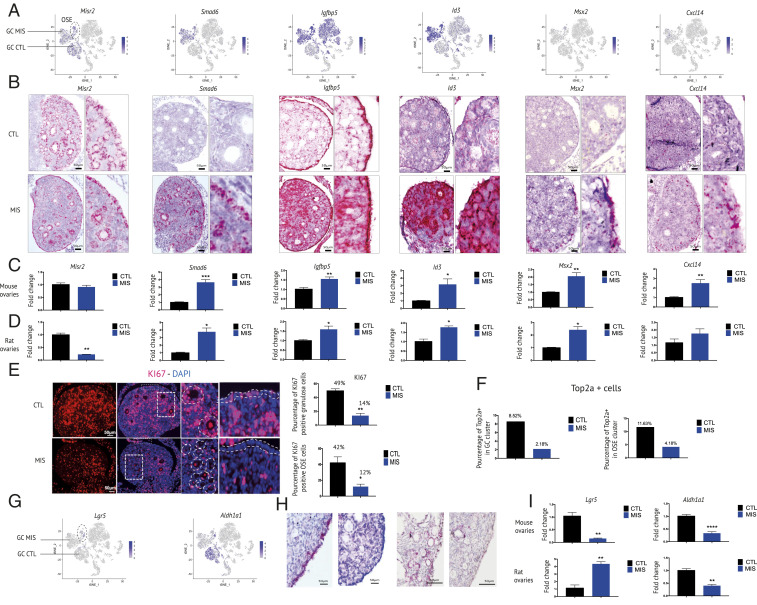
Common gene signatures associated with MIS treatment in ovarian surface epithelium (OSE) and granulosa cells. (*A*) Gene expression levels of representative granulosa cell and OSE markers in *t*-SNE feature plots (arrow refers to granulosa cell, and dashed circles, OSE cluster). (*B*) Expression pattern of granulosa cells and OSE cluster markers by RNAish (*Misr2*, *Smad6*, *Igfbp5*, *Id3*, and *Msx2*) in PND6 mouse ovaries. (*Right*) Magnification of OSE and granulosa cells. (*C* and *D*) RT-qPCR levels of *Misr2*, *Smad6*, *Igfbp5*, *Id3*, and *Msx2* in CTL and MIS PND6 mouse (*C*) and rat ovaries (*D*). The *y* axis shows the fold change normalized to control ovary expression levels. (*≤0.05; **≤0.01, ***≤0.005; unpaired Student’s *t* test, *n* ≥ 3 per group). (*E*) Representative immunofluorescence micrograph of the KI67 (red) proliferation marker in OSE and granulosa cells from CTL- and MIS-treated PND6 rat ovaries. The mean percentage of KI67-positive OSE and granulosa cells was calculated by manually selecting the corresponding areas (surface, follicles) and counting the number of nuclei and Ki67-stained cells. The white dotted lines show representative areas selected for Ki67 quantification, while the complete quantification is presented in the corresponding bar chart (three representative slides from three animals/group). (*F*) Bar chart representing the proportion of Top2a-expressing cells in granulosa and OSE clusters in the scRNA-seq dataset. (*G*) *t*-SNE feature plots of *Lgr5* and *Aldh1a1* expression in response to MIS treatment. The arrows demarcate to granulosa cells from CTL (GC CTL) and MIS-treated animals (GC MIS). The dashed circles show the OSE cluster. (*H*) *Lgr5* and *Aldh1a1* RNAish in PND6 CON and MIS mice ovaries. (*I*) *Lgr5* and *Aldh1a1* qPCRs performed on PND6 mouse and rat ovaries. The *y* axis shows the fold change normalized to control ovary expression levels (**≤0.01, ***≤0.005; unpaired Student’s *t* test, *n* ≥ 3 for CTL and MIS). (Scale bars, 50 μm.)

To evaluate the response of MISR2+ OSE and granulosa cells to MIS, we chose to validate the most prominent (by fold change), statistically significant, differentially expressed markers modulated by MIS administration, as identified in our scRNA-seq dataset (Dataset S1 and *SI Appendix*, Fig. S3 *A*–*D*). We treated mouse and rat pups with AAV9 delivering MIS at PND1 and collected ovaries at PND6 as performed for the scRNA-seq (Fig. 1*D*). Single-cell sequencing showed that OSE cells express markers such as *Misr2*, *Txnip*, and *Lgr5*, which were down-regulated upon treatment, and *Smad6*, *Igfp5*, *Id3*, and *Msx2*, which were up-regulated (Fig. 3 *A*–*D*). These MIS-regulated genes in the OSE were confirmed by RNAish in the mouse (Fig. 3 *B* and *H*), and qPCR both in the mouse and rat ovaries (Fig. 3 *C*, *D*, and *I*). A common set of genes regulated by MIS in the same manner in both the OSE and granulosa cells (*SI Appendix*, Fig. S3*E*) was identified as seen in heatmap (*SI Appendix*, Fig. S3 *A* and *B*), *t*-SNE plots (Fig. 3 *A* and *H*), and RNAish (Fig. 3 *B* and *G*) analyses. In the scRNA-seq dataset, OSE had 61 genes significantly regulated by MIS treatment (*P* < 0.01), while the granulosa cells had 559 (*P* < 0.01) (*SI Appendix*, Fig. S3*E*). Both cell types shared a signature of 39 genes, which included most prominently *Igfbp5*, *Aldh1a1*, *Kctd14*, *Id3*, *Id2*, and *Smad6*; of which the latter three are known canonical targets of MIS (11) (*SI Appendix*, Fig. S3 *C*–*E*). Furthermore, cellular proliferation was inhibited in both granulosa and OSE cells, as evidenced by the reduced proportion of Top2a-positive cells in the scRNA-seq dataset (Fig. 3*F*) and as validated by Ki67 immunofluorescence within the specific layers corresponding to these cell types in ovarian sections (Fig. 3*E*). Of particular interest was the down-regulation of the progenitor markers *Lgr5* and *Aldh1a1* particularly prominent in OSE (Fig. 3 *G*–*I*).

### MIS Inhibits a Granulosa Cell-Specific Differentiation Program.

**Fig. 4.**
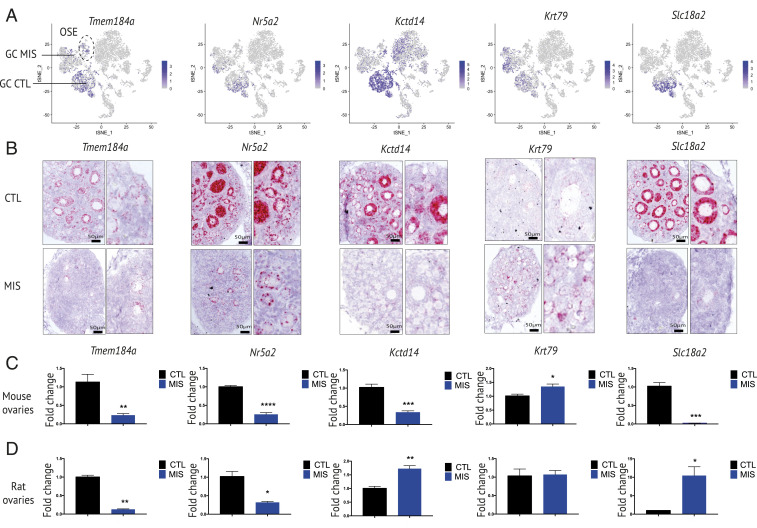
Unique gene signatures in granulosa cells of MIS-treated mice and rats validated in vivo and in vitro. (*A*) Gene expression levels of representative granulosa cell markers overlaid on *t*-SNE plots (arrow refers to granulosa cell). (*B*) Expression pattern of markers specific to granulosa cell cluster (*Tmem184A*, *Nr5a2*, *Kctd14*, *Krt79*, and *Slc18a2*) by RNAish in CTL- and MIS-treated ovaries. *Insets* magnify granulosa cells from the control- and MIS-treated tissues. (*C* and *D*) RT-qPCR levels of *Tmem18A4*, *Nr5a2*, *Kctd14*, *Krt79*, and *Slc18a2* in PND6 mouse (*C*) and rat (*D*) CTL and MIS ovaries. The *y* axis shows the fold change normalized to control ovary expression levels (*n* ≥ 3 for CTL and MIS; *≤0.05; **≤0.01; ***≤0.001; unpaired Student’s *t* test).

While genes like *Smad6* were similarly regulated by MIS in OSE and granulosa (Fig. 3*B*), other down-regulated targets such as *Tmem184a*, *Slc18a2*, *Nr5a2*, or up-regulated genes like *Krt79* were selectively modulated in granulosa cells, as seen on feature plots (Fig. 4*A*), RNAish (Fig. 4*B*), and validated by qPCR (Fig. 4 *C* and *D*). To confirm that these targets were directly regulated by MIS in granulosa cells and not through an effect of MIS on the pituitary-gonadal axis, we performed an experiment in which PND2 rat ovaries were cultured ex vivo in presence of recombinant MIS protein (10 μg/mL) for 48 h on Transwell inserts. Up-regulation of *Id3* and *Igfbp5* upon MIS stimulation was confirmed in granulosa and OSE cells by RNAish (*SI Appendix*, Fig. S4*A*), which was corroborated by qPCR in identically treated samples (*n* = 3), showing significant up-regulation *of Smad6*, *Igfbp5*, and *Id3*, but not of *Nobox*, a germ cell marker (*SI Appendix*, Fig. S4*B*).

### Exogenous MIS Affects Gene Expression in the Ovarian Stroma.

**Fig. 5.**
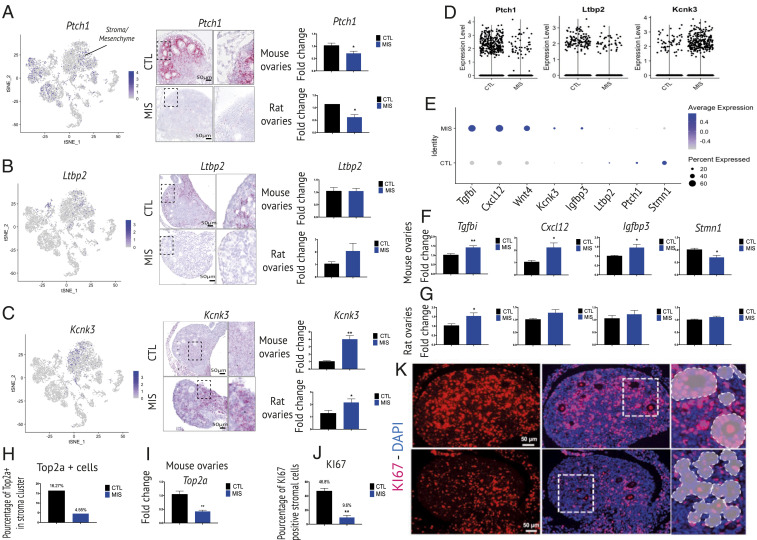
Changes in the gene signatures of the mesenchymal/stromal cells. (*A*–*C*, *Left*) Gene expression levels of representative mesenchymal cells markers, *Ptch1*, *Ltbp2*, and *Kcnk3*, *t*-SNE feature plots in PND6 CTL and MIS mouse ovaries. (*Middle*) RNAish of these three mesenchymal markers. *Insets* in dotted rectangles demarcate to the mesenchymal cells from the control and MIS sections (at *Right*). (*Right*) RT-qPCRs of *Ptch1*, *Kcnk3*, and *Ltbp2* performed on PND6 CTL and MIS mouse and rat ovaries. (*D*) Volcano plots showing expression of *Ptch1*, *Kcnk3*, *Ltbp2*, markers specific to the stroma/mesenchyme, in PND6 mouse CTL- and MIS-treated ovaries based on scRNA-seq data. (*E*) Expression levels and percentages of selected markers in CTL- and MIS-treated mesenchymal cells based on scRNA-seq. (*F* and *G*) RT-qPCRs performed on whole ovaries of *Tgfbi*, *Cxcl12*, *Igfbp3*, and *Stmn1* of mesenchymal/stromal cells markers in the CTL- and MIS-treated mouse (*F*) and rat (*G*) ovaries. The *y* axis shows the fold change normalized to control ovary expression levels (*≤0.05, **≤0.01; unpaired Student’s *t* test, *n* ≥ 3 for CTL and MIS). (*H*) Bar chart representing the proportion of Top2a-expressing cells within the stroma/mesenchyme cluster of MIS-treated and control scRNA-seq datasets. (*I*) qPCR of *Top2a* expression in CTL and MIS PND6 whole-mouse ovaries. (*J*–*K*) Immunofluorescence of Ki67 expression pattern in ovarian stroma/mesenchyme from CTL and MIS rat ovaries. (*J*) Percentage of Ki67-positive mesenchymal/stromal cells in rat ovaries. (*K*) Ki67 immunofluorescence (red). The white dotted lines demarcate stroma/mesenchyme cells, magnified on the *Middle Inset. Right Inset* shows Ki67-positive cells quantification with manual removal of follicles and OSE. (Scale bars, 50 μm.)

While the MIS receptor *Misr2* is primarily expressed by granulosa and OSE cells, we expected to observe indirect (non-cell-autonomous) dysregulation of gene expression in the ovarian stroma given the profound inhibition of folliculogenesis, and the extensive tissue remodeling normally necessary to accommodate this process (25). When comparing differentially expressed markers based on MIS treatment, within the combined mesenchymal supercluster (Fig. 2*B*), we identified a large number of significantly dysregulated genes (by adjusted *P* value), as seen in a representative heatmap (*SI Appendix*, Fig. S5*A*). Of these top markers, we chose to validate subsets of up-regulated (*Tgfbi*, *Igfbp3*, *Cxcl12*, *Kcnk3*) and down-regulated genes (*Stmn1*, *Ptch1*, *Ltbp2*) in the MIS-treated group, based on their specificity as markers of the mesenchyme or their association to MIS treatment in other clusters (Fig. 5). Specifically, the expression of *Ptch1* and *Ltbp2*, which were expressed in the mesenchymal cluster (Fig. 5 *A* and *B*), was down-regulated by MIS treatment in the scRNA-seq dataset (Fig. 5 *D* and *E*), which was confirmed by RNAish and qPCR (Fig. 5 *A* and *B*). Intriguingly, Ltbp2 followed the opposite pattern in OSE cells (up-regulated by MIS; *SI Appendix*, Fig. S5*B*), which could explain why ovary qPCR results were not significantly affected as a whole (Fig. 5*B*), and may indicate that TGFb signaling is differentially regulated in those cells. Some of the pathways known to be directly up-regulated by MIS such as Id2/Id3/Smad6, were paradoxically down-regulated in adjacent cell compartments such as the stroma, presumably in a non-cell-autonomous manner. Others, such as *Kcnk3*, were uniquely up-regulated by MIS in the mesenchyme supercluster, which was also confirmed by RNAish (Fig. 5*C*). The differential expression of *Ptch1* and *Kcnk3* was confirmed by qPCR (Fig. 5 *A* and *C*). Furthermore, MIS treatment was associated with fewer cells expressing cell cycle markers like *Top2a* and Ki67 in the mesenchyme (Fig. 5 *H*–*K*), suggesting MIS treatment resulted in a reduction of proliferation in the stroma. Confirmation by immunofluorescent staining of the Ki67 mitotic marker, showed robustly reduced signal in stromal, granulosa, and OSE cell compartments (Figs. 3 *E* and 5*J*).

### Treatment with MIS Incurs Modest Changes to Oocyte Cell States.

**Fig. 6.**
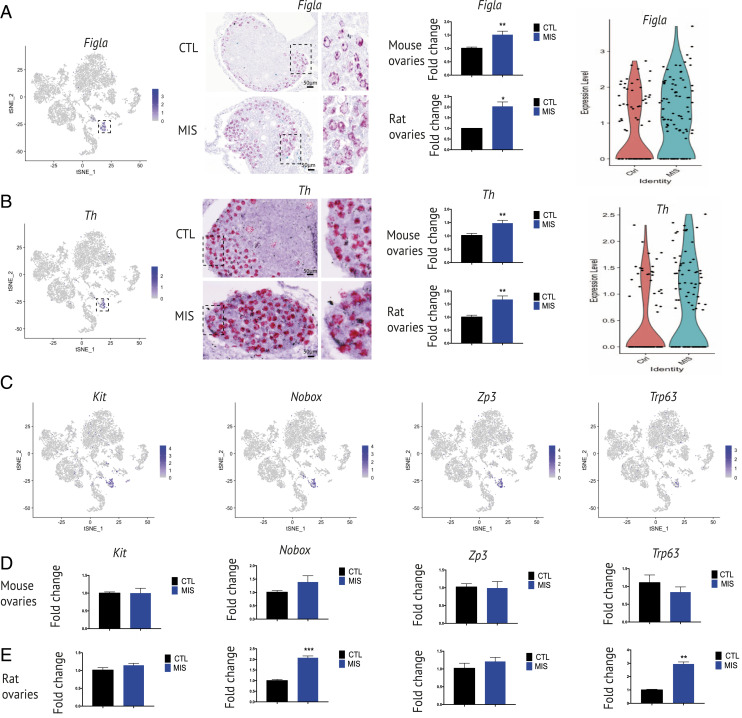
Oocyte response to MIS treatment. (*A* and *B*, *Left*) Gene expression levels of germ cell markers, *Figla* and *Th* in *t*-SNE feature plots of PND6 CTL and MIS mouse ovaries, in PND6 CTL and MIS mouse ovaries. The dotted circles and black arrows demarcate the oocyte cluster. (*Middle*) RNAish of these same markers. (Scale bars, 50 μm.) *Insets* in dotted rectangles demarcate the oocytes from the control and MIS-treated sections. (*Right*) RT-qPCR of these same markers in both PND6 mouse and rat ovaries. Violin plots from scRNA-seq data. (*C*) Gene expression levels of germ cell markers *Kit*, *Nobox*, *Zp3*, *Trp63*, on *t*-SNE feature plots in PND6 CTL and MIS mouse ovaries. The dotted square demarcates the germ cells/oocytes cluster. (*D* and *E*) RT-qPCR of oocytes markers, *Kit*, *Nobox*, *Zp3*, *Trp63*, qPCRs performed on PND6 mouse (*D*) and rat (*E*) ovaries. The *y* axis shows the fold change normalized to control ovary expression levels (*n* ≥ 3 for CTL and MIS).

In contrast to other cell types such as granulosa, OSE, and stromal cells, MIS had a more modest effect on the gene expression profile of the oocytes recovered in our scRNA-seq dataset, although our ability to detect these perturbations was limited by the lower recovery of these cells. Indeed, only three genes were significantly differentially expressed by adjusted *P* value: *Hist3h2a*, *Ubb*, and *Gm9493* (*SI Appendix*, Fig. S6*B*). The oocytes were identified using accepted germ cell markers such as *Figla* and Th, which appear highly specific to the germ cell cluster and were validated by RNAish (Fig. 6 *A* and *B*). The relative expression of germ cells markers like *Kit* and *ZP3* was not affected by treatment with MIS (Fig. 6 *C–E*) by scRNA-seq, which was confirmed by qPCR. In contrast, the up-regulation trends of *Figla*, *Th*, and *Nobox* following AAV9 MIS administration observed in the scRNA-seq data became significant when validated by qPCR. The up-regulation of these genes could reflect the higher proportion of quiescent primordial follicles versus activated follicles (26), and the higher relative abundance of primordial follicles within the total ovarian cell types given the inhibition of OSE, and stromal compartment in MIS-treated ovaries (Fig. 6*E*). This latter interpretation was consistent with the increased density of Ddx4-positive germ cells, as seen in representative ovarian sections stained by immunofluorescence (*SI Appendix*, Fig. S6*A*). Moreover, when measuring oocyte diameters on whole-mount DDX4-stained ovaries (*SI Appendix*, Fig. S6 *C* and *D*), we confirmed an overall decrease in mean oocyte size as well as a decrease in the population of larger oocytes that are present in secondary follicles, which is consistent with total follicle counts through serial sectioning of PND15 rat ovaries (Fig. 1*I*).

To explore the potential effect of MIS on cell–cell communication within the follicle, we employed the CellPhone DB algorithm, which we had previously adapted for use with mouse/rat datasets (11). This analysis allowed us to identify significant ligand/receptor pairing interactions and their direction as autocrine or paracrine signals in granulosa cells and oocytes. By contrasting this analysis in control and MIS-treated ovaries, we identified signals unique to the MIS treatment, or missing in the MIS treatment, which may underlie the cell-autonomous and non-cell-autonomous effects of MIS in the follicle. Strikingly, we found multiple alterations in TGF-beta pathways, such as autocrine TGFB2–TGFBR3 and TGFB3–TGFBR3 interactions uniquely present in MIS-treated granulosa cells while other paracrine signals such as BMP4–BMPR2 from granulosa to oocytes were absent in treated ovaries (*SI Appendix*, Fig. S5*D*). Other examples of pathways uniquely present in MIS treatment include FGF9–FGFR2 signaling from the oocyte to the granulosa and EFNB1–EPHA4, aVb5–FN1, and aVb1–FN1 from the granulosa to the oocyte, which may underlie non-cell-autonomous or indirect effects of MIS on the follicle (*SI Appendix*, Fig. S5*D*).

### MIS Maintains Granulosa Cell Quiescence and Prevents Normal Development of Activated Follicles.

**Fig. 7.**
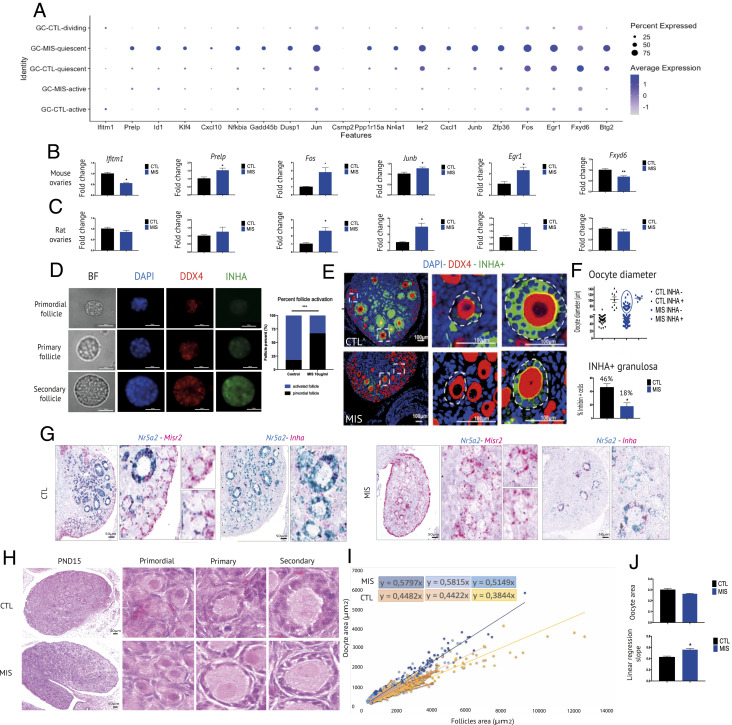
MIS maintains granulosa cell quiescence and prevents normal development of activated follicles. (*A*) Expression levels and percentages of quiescence-related markers in control- and MIS-treated granulosa cells based on their state of activation. (*B* and *C*) RT-qPCR validation of quiescence genes performed on PND6 CTL and MIS mouse (*B*) and rat (*C*) ovaries. *y* axis shows the fold change normalized expression levels relative to control ovary (*<0.05, **<0.01; unpaired Student’s *t* test, *n* > 3 for CTL and MIS). (*D*) Micrographs of brightfield, and DAPI, DDX4, or INHA immunofluorescence of representative primordial, primary and secondary follicles dissected from adult mouse ovaries. Percentage of primordial and activated follicles following 24 h of culture with control or 10 μg/mL MIS. (*E*) Double immunofluorescence DDX4 (red)/INHA (green) of PND6 CON and MIS rat ovaries. Examples in *Insets* of normal INHA+ growing preantral follicles with one or several layers of cuboidal granulosa cells (*Up Middle*, *Right*, circled with white dashes), and MIS-treated abnormal follicles with large oocytes surrounded by inhibin negative squamous granulosa cells or singular layer of weakly positive INHA+ granulosa cells (*Down Middle*, *Right*, circled with white dashes). (*F*) Oocyte diameter in follicles of CTL- and MIS-treated PND6 rat ovaries classified according to the INHA immunofluorescence status of their surrounding granulosa cells (*Up*). A population of large oocytes (diameter, >66 μm; smallest activated primary follicles in CTL) surrounded by INHA-granulosa cells in MIS-treated ovaries are circled in blue. Percentage of INHA+ granulosa cells surrounding growing large oocytes (diameter, >66 μm) (*Down*). (*G*) RNAish showing colocalization of *Misr2* (red)/*Nr5a2* (green) and *Nr5a2* (green)/*Inha* (red) in PND6 CTL- and MIS-treated mouse ovaries. (Scale bars, 50 μm.) *Insets* magnify follicles from CTL and MIS-treated tissues. (*H*) Histology of H&E-stained 5-μm section of CTL- and MIS-treated rat ovaries at PND15. Representative images of primordial, primary and secondary follicles are shown in *Insets*. (Scale bars, 50 μm.) (*I*) Scatterplot of oocyte area by function of follicle area with linear regression in PND6 MIS-treated and CTL mouse ovaries (*n* = 3). (*J*) Average oocyte diameter in PND6 mice ovaries (*Up*). Average linear regression slope from Fig. 7*I* (*Down*).

To explore further the effect of MIS on early follicular development, we compared the gene expression signatures of the five granulosa cell subclusters identified within our dataset. All clusters expressed typical granulosa cell markers such as *Nr5a1*, *Fst*, and *Amhr2*; three clusters were composed almost entirely of control cells (GC-CTL-quiescent, GC-CTL-active, GC-CTL-dividing) and two mainly of MIS cells (GC-MIS-quiescent, GC-MIS-active) (*SI Appendix*, Fig. S7*A*). Dividing granulosa cells in the control group had a characteristic cell cycle signature, which included *Top2a*, *Tk1*, and *Stmn1*, and likely corresponded to growing primary to secondary follicles, which were otherwise suppressed in the MIS-treated group (*SI Appendix*, Fig. S7*A*). The “active” granulosa cell cluster present in the control group was defined by its expression of classic markers of early follicular growth, such as *Inha*, *Inhb*, *Slc18a2*, and *Hsd3b1* (27), while the “quiescent” cluster had low expression of these same markers (*SI Appendix*, Fig. S7*A*). The two clusters found in the MIS-treated group largely recapitulated this relationship and were called quiescent and active, but were different in two important ways: the GC-MIS-active cluster had generally lower expression of the differentiation markers (*Inha*, *Inhb*, *Slc18a2*, and *Hsd3b1*) when compared to the GC-CTL–active cluster (*SI Appendix*, Fig. S7), and both the GC-MIS–active and GC-MIS–quiescent clusters harbored the distinctive MIS pathway gene signature, which included up-regulation of *Id2*, *Id3*, *Smad6*, and *Igfbp5* (*SI Appendix*, Fig. S7*A*). The relatedness of all five granulosa cell clusters was further elaborated by comparing average gene expression in each cluster and producing a phylogenetic cluster tree which confirmed that the active and “dividing” clusters of the control group were the most closely related, and that the quiescent control cluster segregated with the MIS clusters (*SI Appendix*, Fig. S7*B*), suggesting MIS suppression induced a transcriptional program similar to quiescence. The transcriptional signature associated with the quiescent state included genes such as *Btg2*, *Fxyd6*, *Egr1*, *Fos*, *Zfp36*, *Junb*, *Cxcl1*, *Ier2*, *Nr4a1*, *Ppp1r15a*, *Csrnp2*, *Jun*, *Dusp1*, *Gadd45b*, *Nfkbia*, *Cxcl10*, *Klf4*, *Id1*, *Prelp*, and *Ifitm1* (Fig. 7*A*). We then confirmed regulation of these markers by MIS by qPCR in both PND6 rat and mouse ovaries and showed up-regulation of proquiescence markers such as *Fos*, *Junb*, or *Egr1* and down-regulation of *Ifitm1* and *Fxyd6* (Fig. 7 *B* and *C*). To confirm further that MIS directly maintains primordial follicles in a quiescent state, we used a bioassay in which we cultivated individual primordial follicles with MIS or control and showed a significant decrease in the rate of activation, as confirmed by the decreased proportion of INHA-positive follicles (Fig. 7*D*).

To test the hypothesis that MIS induced a transcriptional profile that imposed quiescence in primordial follicles, but also suppressed differentiation of granulosa cells in “activated” follicles, we performed a phenotypic analysis of follicles with growing oocytes. To do so, we treated rats with AAV9-MIS or AAV9-control (*n* = 3/group), and stained the middle sections of the ovary for DDX4 (Vasa) as a marker of germ cells, and INHA as a marker of active granulosa cells by immunofluorescence (Fig. 7*E*). The appearance of follicles with large oocytes differed in MIS-treated ovaries compared to controls; we observed oocytes of size expected in primary follicles but containing squamous granulosa cells, or even the size expected in secondary follicles but surrounded by INHA-granulosa cells (Fig. 7*E*), suggesting developmental delays in granulosa cells. This phenotype was quantified by follicle morphometrics, demonstrating that MIS resulted in a statistically significant decrease in INHA^+^ staining in granulosa cells surrounding large-diameter growing oocytes (diameter > 66 μm, the lower threshold observed for INHA^+^ follicles in controls), with a corresponding appearance of a population of oocytes (circled) within follicles devoid of INHA^+^ cells (Fig. 7*F*). To examine further the expression of maturation markers in quiescent and activated primordial follicles after MIS treatment, we performed in situ colocalization of Inha with both Misr2 as a marker of primordial follicles, and Nr5a2 as a marker of granulosa cell activation (28), which identified activated primordial follicles in the cortex of control ovaries (Misr2+/Inha+, Nr5a2+/Inha+), but not in MIS-treated ovary, where this combination was found almost exclusively in large medullary follicles with a single layer of granulosa cells (Fig. 7*G*).

Histological evaluation of the rat ovaries at PND15 confirmed this characteristic pattern in the MIS-treated group, with normal histology of primordial follicles, yet abnormal “primary” follicles, which contained disproportionately large oocytes, and “secondary” follicles, which displayed abnormally fewer layers of granulosa cells with a more squamous appearance compared to the matched controls (Fig. 7*H*). These data suggest a defect in granulosa cell proliferation and an inhibition of follicle development independent of oocyte growth (Fig. 7*H*). To confirm that MIS suppression in folliculogenesis is not driven by inhibition of oocyte growth, but rather the desynchronization of maturation of granulosa cells, we examined the size of oocytes following AAV9 MIS administration at PND1. This analysis showed no significant difference between control and MIS-treated mice at PND6, despite a trend for smaller diameter consistent with inhibition of primordial follicle activation (Fig. 7 *I* and *J*). We further characterized follicular growth by measuring both oocytes and their corresponding follicle areas in six nonconsecutive representative ovarian sections (*n* = 3 mice/per group) of PND6 AAV9 MIS or AAV9 empty control-treated mice. Importantly, MIS treatment resulted in a reduced rate of growth of follicle area as a function of oocyte diameter growth (Fig. 7 *I* and *J*). This finding was interpreted to indicate that if, despite suppression, a primordial follicle does undergo activation, MIS continues to inhibit granulosa cell differentiation and proliferation during preantral stages, suggesting MIS plays an inhibitory role that extends beyond activation in neonatal ovaries.

## Discussion

Female mammalian gonads are endowed with an allotment of follicles during the perinatal period, which is expended over the reproductive life span. As we begin to appreciate better the complex events occurring during the embryonic and early postnatal formation of primordial follicles, it becomes essential to identify the factors regulating their entry into long-term quiescence and their activation. MIS/AMH, produced by the granulosa cell of early growing follicles, has been described as an inhibitor of germ cyst breakdown (3) and primordial follicle activation (15, 20, 29). Herein, we define the cellular atlas and transcriptional changes associated with MIS suppression of folliculogenesis by scRNA-seq during the first wave of primordial follicle formation, activation, and growth in mice at PND6. When evaluating the different populations of follicles in PND15 rat ovaries, we confirmed previous observations in the mouse (10) with a trend for an increase in primordial follicles along with the dramatic significant decrease in the number of primary, secondary, and antral follicles. The lack of statistical significance in the excess primordial follicles, as expected from an inhibition of activation, could be the result of a smaller initial size of the primordial follicle pool, as reported by studies that describe inhibition of germ cyst breakdown by MIS (3). The timing of our study coincides exposure to MIS (PND1–6) with the formation of primordial follicles, and their initial activation and growth (2). Moreover, the continued expression of early germ cell markers like *Figla* in the AAV9 MIS-treated mice may reflect this inhibition of primordial follicle assembly (3).

Our data demonstrate that MISR2 is expressed as early as E19.5 in pregranulosa cells and OSE, and continues to be expressed in those cell types postnatally, including in granulosa cells of all follicle types, a pattern that may reflect the common embryonic origin of OSE and granulosa cells (30, 31). Indeed, these cell types may respond in a similar fashion to MIS, as our scRNA-seq dataset revealed 39 commonly MIS-regulated genes in OSE and granulosa, including canonical targets of MIS such as *Misr2*, *Id2*, *Id3*, *Smad6*, and *Igfbp5* (11) as well as a number of novel target genes such as *Kctd14* and *Aldh1a1*. MIS may regulate multiple TGFB pathways, as suggested by its up-regulation of the inhibitory Smad6/7, and down-regulation of Smad3, and potential effects on BMP4 signaling between the granulosa and oocyte. Given the data presented herein, it is likely that MIS regulates a large number of pathways both directly in granulosa and OSE cells, but also indirectly in oocytes and stromal cells that did not express Misr2.

Ultimately, proliferation of granulosa, OSE, and stromal cells was similarly down-regulated by MIS. The reduced proliferation of OSE was accompanied with a decrease in Aldh1a1 and Lgr5, which had been proposed as markers of OSE progenitors (31–33), although we found these markers to be broadly expressed in the OSE of control ovaries at PND6. Lgr5 has recently been showed to be expressed in the epithelial pregranulosa, a subgroup of pregranulosa cells that predominate in the cortex and differentiate into granulosa cells of quiescent follicles, constituting the second wave of primordial follicles in charge of sustaining fertility (34). It is unclear whether the mechanisms regulating primordial follicle activation and subsequent growth differ between these two pools, although both types of primordial follicles are presumably represented in our single-cell transcriptomic dataset.

Compared to the transcriptional effects of MIS in somatic cells in the ovary, the germ cells, which do not express MISR2, were relatively unchanged by MIS. Interestingly, both Th, specific to oocytes, and Slc18a2, a monoamine transporter, which was specific to granulosa cells, were significantly transcriptionally regulated by MIS (up and down, respectively). Together, these genes represent essential and rate-limiting components of the monoamine biosynthesis pathway, suggesting a possible MIS-dependent role of this class of neurotransmitters in folliculogenesis (35, 36).

Although multiple ovarian cell types responded to MIS treatment, granulosa cells had the most profound transcriptional response, permitting separation of control and treated granulosa cell clusters in *t*-SNE plots. These could be further subdivided into five different subclusters, seemingly recapitulating the state of activation/repression of the granulosa cells. This led us to investigate quiescence-associated markers that were regulated by MIS. As part of this signature, we showed an up-regulation of *Egr1*, *Junb*, and *Txnip*, which had been previously reported as proquiescence markers in hematopoietic cells (37), as well as a down-regulation of markers known to be associated with granulosa cell differentiation, activation, and proliferation, such as *Inha* and *Nr5a2* (28). In a recent study, we have reported that *Nr5a2* conditional knockout in granulosa cells leads to a drastic decrease in the number of primary follicles, suggesting its expression is necessary for the full activation of the primordial follicle (28). This observation was supported by the marked decrease of Nr5a2-positive primordial follicles following MIS treatment observed herein.

Other studies have shown that *Inha*^−/−^ ovaries develop secondary follicles with an abnormally large number of granulosa cell layers as well as an increased frequency of early antral stage follicles, suggesting a dysregulation of the communication between germ cells and granulosa cells and excess growth (38). Conversely, MIS treatment may antagonize granulosa cell activation, differentiation, and growth, inhibit Inha and Nr5a2 expression, and result in the uncoupling of oocyte and granulosa maturation, with the former expanding without proliferation of the latter. The synchronization between granulosa cells and oocyte activation is essential for the success of follicle development. This is evidenced by studies suggesting inhibition of Kit ligand in granulosa cells or Kit receptor in the oocyte of primordial follicles prevents their differentiation by maintaining oocyte quiescence, while overexpression of mTORC1 accelerates said differentiation leading to premature activation of all dormant oocytes and primordial follicles (39–41). A parallel can also be made between the MIS phenotype described herein, and a mouse model in which expression of constitutively activated kit induces PI3K signaling in oocytes and specifically overrides oocyte quiescence, leading to rapid growth and depletion of all oocytes, often in follicles with similarly undifferentiated granulosa cells (27). These data suggest that both granulosa cells and oocytes may play a role in the initial activation of primordial follicles, and that coordinated activation may be necessary for successful maturation of the follicle (42). Seemingly, neither forced activation through the oocyte nor induced quiescence of granulosa cells alone may completely control the fate of a follicle.

Elucidating the mechanism of inhibition of follicular development by MIS will help us to identify novel therapeutic targets that could modulate the ovarian reserve. In granulosa cells, treatment with MIS may suppress a number of parallel growth pathways, which might otherwise converge with activation. A number of factors identified herein are secreted and eminently druggable, such as the IGF modulating binding proteins Igfbp5 and Igfbp3, or the cytokines Cxcl1 and Cxcl14 (43) represented in the quiescence signature, and their autocrine and paracrine effects on primordial follicle quiescence should be further investigated. We also identify a number of genes known to mediate germ cell–somatic cell communication and differentiation, such as TMEM184A, which is expressed in Sertoli cells and whose ectopic expression in female genital ridge engenders a failure of gonocytes to enter meiosis and caused XX germ cells to commit to the male fate (44, 45). Finally, the strong enrichment of factors involved in immediate-early gene response and Nfkb signaling in the quiescent granulosa cell may open up further studies to identify the precise downstream signaling mechanism and transcription factors responsible for the maintenance of the long-term quiescence program.

Altogether, we have shown that exogenous MIS treatment affects multiple cell types of the neonatal ovary likely via both autonomous and non-cell-autonomous mechanisms. The most conspicuous effect of MIS was observed in granulosa cells whose activation and differentiation were prevented by MIS despite continued growth of the oocyte in a small subset of follicles. These findings suggest that MIS may be an effective ovarian suppressant, capable of inhibiting both primordial follicle activation and preantral follicle maturation.

## Materials and Methods

### Animals.

This study was performed according to experimental protocols 2009N000033 and 2014N000275, approved by the Massachusetts General Hospital Institutional Animal Care and Use Committee with Sprague–Dawley rats (purchased from Envingo) and C57 black-6 (C57BL6) mice (purchased from Charles River Laboratories).

### AAV9-MIS Treatment.

A single dose of 2.5e10vp of adeno-associated virus serotype 9 (AAV9) gene therapy vector was injected to PND1 mice and rats for continuous delivery of a high concentration of human MIS analog (albumin leader Q425R MIS or LR-MIS) as previously described (23).

### Estimation of Ovarian Volume.

An estimate of CTL- and MIS-treated ovaries volume at PND6 was obtained using the following formula:

$$V=\frac{1}{2}\left(w^{2}\times l\right),$$

with V, volume; l, length; and w, width of the ovary.

### Immunofluorescence and Immunohistochemistry.

Embedded tissues were sectioned, rehydrated, boiled in sodium citrate, and blocked in 5% BSA/0.1% PBS Tween, incubated overnight with the antibodies of interest (mouse anti-human inhibin alpha [INH] [1:250; Bio-Rad; #MCA951ST], Vasa [1:1,000, Abcam #AB207710], and Ki67 [1:200; ab16667]) before being treated with fluorescent secondary antibodies (Alexa Fluor 555-conjugated donkey anti-rabbit IgG antibody; #A31572; Alexa Fluor 488-conjugated donkey anti-mouse IgG; 1:500), and finally counterstained with DAPI (1:1,000 in PBS; Thermo Fisher; #D1306).

### Histomorphological Analysis of Ovaries.

INHA immunofluorescence images were used to calculate the percentage of mature (cuboidal) granulosa cells in control and MIS-treated ovaries. CellProfiler Software was used for automated cell counts. DDX4 was used to measure the oocyte diameters in control and MIS treated ovaries. Oocytes larger than 66 μm in diameter were considered to be activated (46) and the number of granulosa cells surrounding the said oocytes was quantified using DAPI staining. Inhibin-positive cells were then quantified based on double-positive DAPI/Inhibin signal. Ki67-positive cells were counted using CellProfiler software in manually delimited granulosa cells, stroma, and OSE areas.

Serial H&E sections were used to quantify and classify follicles in rat ovaries, and whole-mount immunofluorescence with DDX4 staining was used in mice to quantify the total number of germ cells in mouse ovaries.

### RNA In Situ Hybridization and Colabeling Experiments.

RNAish was performed with the manual RNAscope probe hybridization according to the manufacturer’s instructions (47). Pre- or custom-designed probes spanning mRNAs of the target genes are listed in *SI Appendix*, Table S2.

### Quantitative PCR.

Total RNA was extracted from two ovaries of control and AAV9-MIS PND6 treated mice or rats using the Qiagen RNA extraction kit, and cDNA was synthesized from total RNA using SuperScript III First-Strand Synthesis System for RT-PCR (Invitrogen; #18080-051). Expression levels relative to the geometric mean housekeeping genes were calculated by using cycle threshold (Ct) values logarithmically transformed using the 2^−ΔCt^ function. Fold changes were calculated relative to PND6 control. Primers sequenced for mouse and rat samples are recapitulated in *SI Appendix*, Tables S3 and S4.

### Western Blot.

Western blots were performed as previously described (*SI Appendix*) using p-Akt (1:500; Cell Signaling; #S473); p-Smad 1/5/8 (1:1,000; Cell Signaling; #9516; lot11) ; and β-actin antibodies (1:5,000; Cell Signaling, #12413). Anti-rabbit IgG HRP-linked was used as secondary antibody (1:2,000; Cell Signaling; #7074).

### ELISA.

ELISA was used to determine AAV9-MIS (human) concentration in mouse and rat serum using in-house-produced anti-MIS antibody, PBS/2% BSA solution as blocking medium, LR-MIS as standard, rabbit polyclonal anti-MIS antibody (MGH6), and a secondary HRP-labeled antibody before being revealed by enzyme substrate (see *SI Appendix, Materials and Methods* for more details).

### Generation of Single-Cell Suspension for scRNA-seq.

Newborn mice (PND1) were injected with 2.5E10 particles of AAV9-MIS (*n* = 3) or AAV9 empty particle controls (*n* = 3). On PND6, mice pups were killed, and the ovaries harvested. Ovaries (*n* = 3 per group) were combined and placed in dissociation medium for single-cell digestion as previously described (11).

### scRNA-seq (inDrop).

inDrop microfluidic sorting was performed as previously described (48) generating two libraries of ∼5,000 cells from each combined (*n* = 3 mice) cell suspension (control and MIS). Transcripts were processed as previously described, and samples were sequenced on a NextSeq 500 (Illumina) in a single combined lane (48).

### Data Analysis of scRNA-seq.

The analysis of the demultiplexed data were performed using the Seurat package in R (24). Filtering parameters were set to remove cells with fewer than 400 genes from the dataset, data were log normalized, and nUMI were regressed. The final dataset included 5,002 control cells and 4,435 MIS-treated cells, which were jointly analyzed. Principal-component analysis was performed using 15 dimensions, and FindClusters parameters were set at a resolution of 0.6.

### CellPhoneDB Analysis.

We predicted signaling between cell clusters through analyzing coexpression of known human receptors and secreted proteins using CellPhoneDB. Ortholog information for the mouse (GRCm39) and human (GRCh38.p12) were obtained from ENSEMBL (release 95). For non-one-to-one orthologs, we assigned the maximum of gene expression values for all mouse to human mappings. CellPhoneDB was subsequently run using default parameters (49).

### Enrichment Analysis.

Differentially expressed genes with at least twofold changes between the MIS-treated granulosa cells and controls were used as input for Gene Ontology Enrichment Analysis by clusterProfiler (50). Enrichplot package is used for visualization (51). Biological process subontology was chosen for this analysis.

### Statistical Analysis.

Unpaired Student’s *t* test was used to compare the control and the treated samples using the Prism software (GraphPad, version 8.0).

## Supplementary Material

Supplementary File

Supplementary File

## Data Availability

Single-cell sequencing data have been deposited in the Open Science Framework (OSF) (https://osf.io/3sbtp/) (52).
