# Supplementary material for: Single-cell sequencing reveals suppressive transcriptional programs regulated by MIS/AMH in neonatal ovaries
Source: Proc Natl Acad Sci U S A. 2021 May 12;118(20):e2100920118. doi: 10.1073/pnas.2100920118 (PMC8157966; doi:10.1073/pnas.2100920118)
Supplement: Supplementary File [file pnas.2100920118.sapp.pdf]

PNAS
www.pnas.org

Supplementary Information for

Single-cell sequencing reveals suppressive transcriptional programs regulated by MIS/AMH in neonatal ovaries.

Meinsohn, MC[1,2], Saatcioglu HD[1,2], Lina Wei[1,2], Yi Li[1,2], Horn H[1,2,3], Chauvin M[1,2], Kano M[1,2], Nguyen NMP[1,2], Nagykery N[1,2], Kashiwagi A[1,2], Wesley R. Samore[4], Wang D[5], Esther Oliva[4], Gao G[5], ME Morris[6], Donahoe PK[1,2], Pépin D[1,2].

[1] Pediatric Surgical Research Laboratories, Massachusetts General Hospital, Boston, United States

[2] Department of Surgery, Harvard Medical School, Boston, United States.

[3] Stanley Center, Broad Institute of MIT and Harvard, Cambridge, United States.

[4] Department of Pathology, Massachusetts General Hospital, Boston, United States.

[5] Horae Gene Therapy Center, University of Massachusetts, Worcester, United States.

[6] Department of Gynecology, Massachusetts General Hospital, Boston, United States.

Corresponding authors: Donahoe PK, Pépin D
Emails: PDONAHOE@PARTNERS.ORG, dpepin@mgh.harvard.edu

This PDF file includes:

Supplementary Material and methods
Figures S1 to S7
Tables S1, S2, S3, S4
Legends for Datasets S1 and S2
SI References

Other supplementary materials for this manuscript include the following:

Dataset S1
Dataset S2

Supplementary Information Text

Material and methods

Ovarian cultures and recombinant MIS protein

Recombinant MIS production was performed as previously described (1, 2). Postnatal day 2 (PND2) mouse or/and rat full ovaries were harvested and placed on trans-well permeable supports (Costar) and were cultured (37C, 5% CO_2) for 48 hours in DMEM-F12 media supplemented with 0.1% Albumax (Thermo Fisher Scientific), penicillin-streptomycin (Invitrogen), 0.1% FBS (Thermo Fisher Scientific), 27.5 µg/ml transferrin (Sigma-Aldrich, Saint-Louis, MO), and 0.05 mg/ml L-ascorbic acid (SigmaAldrich) (3),(4). The ovaries were treated either with mock MIS, recombinant MIS (10ug/ml), or human C-X-C Motif Chemokine Ligand 14 (hCxcl14) recombinant protein (100ng/ml) (R&D systems). Two ovaries were analyzed per condition with at least 3 replicates. Total RNA was isolated from the cultured ovaries and various gene levels were analyzed by qPCRs. Primer sequences are presented in **Tables S3 and S4**.

Whole-mount Immunofluorescence (IF)

Mice were treated with empty AAV9 vector or with AAV9-MIS on postnatal day 1 (PND1) (2.5e10) and sacrificed on day 6 ($n \geq 3$), or harvested at PND2 and treated for 48h with 100ng/mL, before being fixed overnight in 10% formalin. Whole mount immunofluorescence protocol was adapted from the protocol previously described by Rinaldi et al. (5). Briefly, following PBS washes and permeabilization using PBS/1% Triton for 4h, tissues were blocked for 1h in 10% BSA/PBS 1% Triton overnight. The oocyte marker DDX4 (abcam #AB207710) staining was performed (1:1000 in blocking solution) for 4 days and tissue was washed in PBS 1% Triton 3x10 minutes before applying secondary antibody (Alexa Fluor 555-conjugated donkey anti-rabbit IgG antibody, # A31572) for 2 days (1:500 in blocking solution). Finally, samples were placed in clearing solution (2.5ml glycerol/10g sorbitol/5ml DMSO/12ml of 9M urea). The solution was replaced twice a day, for 5 days before mounting.

ELISA

ELISA was used to determine AAV9-MIS (human) concentration in mouse and rat serum. A 96-well high protein-binding capacity plate (Nunc MaxiSorp) was coated with 5µg/ml of in house produced mouse anti-MIS antibody (6E11) overnight at 4°C. Then, the plate was washed 3 times with PBS/0.01% Tween-20 and saturated with a PBS/2% BSA solution for 2hr at room temperature. The LR-MIS standard was added at 35ng/ml and 8 serial 1:2 dilutions were made before adding serum samples previously diluted at 1:100 for 2h. Rabbit polyclonal anti-MIS antibody (MGH6), was added at 1:4 000 and incubated for 2hr. Then a secondary HRP labeled antibody (Donkey anti-rabbit IgG conjugated HRP; Jackson ImmunoResearch Laboratories) was diluted at 1:70 000 and added during 1hr. Lastly, plate was washed five times and revealed by enzyme substrate (TMB). Absorbance was read at 450 nm after stopping the enzymatic reaction by the addition of sulfuric acid. To validate our ELISA, MIS ELISA kit from Beckman and coulter (AMH Gen ELISA kit; ref : A73818) test was realized in parallel according the manufacturer protocol.

Western blot

Protease inhibitor cocktail (Roche Indianapolis, IN) and phosphatase inhibitor tablets (Roche, Indianapolis IN) were used to prepare the total protein lysates. Equal amounts of reduced protein (15ug) were loaded in each well after protein quantification by Pierce bicinchoninic acid assay (BCA) protein quantification kit (Thermoscientific, Rockford IL). Proteins were transferred to Immobilon polyvinylidene difluoride (PVDF) membranes (Millipore, Billerica MA), blocked in 5% non-fat milk dissolved in a tris-buffered saline and tween 20 (TBST) solution, and primed with p-Akt (1:500; Cell Signalling #S473); p-Smad 1/5/8 (1:1000; Cell Signalling, #9516, lot11) ; and β-actin antibodies (1:5000; Cell signaling, #12413). Anti-rabbit IgG HRP-linked was used as secondary antibody (1:2000; Cell signaling, #7074). ProteinSimple FluorChem Western Imaging System was used for imaging. Quantification of Western blots was realized based on three independent protein extractions. Intensity of each protein band was measured using ImageJ software and rationalized on the housekeeping protein (β-actin).

Immunofluorescence (IF) and immunohistochemistry (IHC)

Dissected ovaries or embryos were fixed in 4% (wt/vol) paraformaldehyde at 4 °C (for embryos histology) or in 10% neutral buffered formalin at room temperature overnight (for ovarian histology, RNAish and immunofluorescence). Tissues were embedded in paraffin blocks in an automated tissue processor (Leica #TP1020). 5µm ovarian sections were used for hematoxylin and eosin (H&E) staining, immunofluorescence (IF), and RNAish using the RNA scope™ (ACD Bio) system. Human ovarian blocks were procured by the Massachusetts General Hospital, Gynecological Pathology Department through an IRB approved protocol (IRB 2007P001918) (6).

For immunofluorescence (IF) experiments, tissue sections were rehydrated in an alcohol series after deparaffinization in xylene. Antigen retrieval was performed by parboiling in 10 mM sodium citrate (pH 6.0), cooling at room temperature, blocking in 5% bovine serum albumin (BSA) in Phosphate-buffered solution (PBS)/0.1% Tween 20 for one hour at room temperature, and incubated with primary antibodies overnight at 4°C ((Mouse anti-human inhibin alpha (INH) (1:250, biorad #MCA951ST), Vasa (1:1000, abcam #AB207710), Ki67 (1:200, ab16667)). The slides were blocked and incubated in fluorescently conjugated secondary antibodies (Alexa Fluor 555-conjugated donkey anti-rabbit IgG antibody, # A31572; Alexa Fluor 488-conjugated donkey anti-mouse IgG, - 1:500), counterstained for 5mn with DAPI (1:1000 in PBS, ThermoFisher #D1306) and cover-slipped with vectashield mounting medium (Vector Laboratories # NC9265087). For immunohistochemistry (IHC), Dako EnVision+ System- horseradish peroxidase (HRP) Labelled Polymer Anti-Rabbit was used as the secondary antibody (#K4002), and the HRP signal was detected using the DAKO detection system (Dako, #K5007).

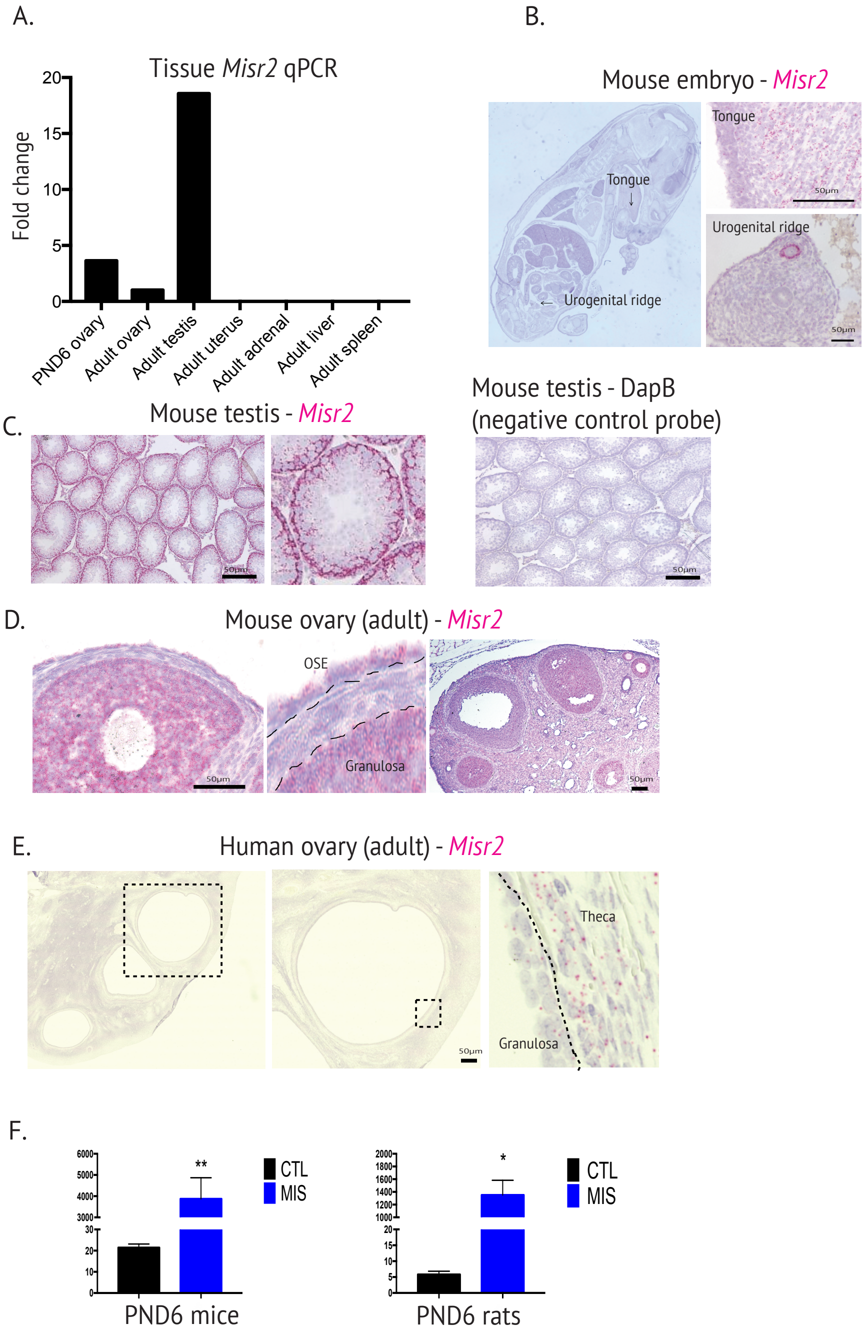

Fig. S1. Misr2 expression pattern in tissues. A. RT-qPCR levels of *Misr2* expression pattern among different tissues in mice (PND6 ovaries, adult ovaries, uterus, liver, testis and adrenal). **B**. *Misr2* expression pattern in sagittal sections of E17.5 mouse embryos by RNAish. Tongue and

urogenital ridge tissues are shown in the inserts. **C.** *Misr2* and *DapB* expression pattern on mouse testes (3 weeks old) by RNAish. *DapB* (*E. coli* dihydrodipicolinate reductase) probe was used as negative control (right panel). Insert shows specific *Misr2* expression in Sertoli cells from a seminiferous tubule. **D.** *Misr2* expression pattern in adult mouse ovary shown by RNAish (4 weeks old). **E.** *Misr2* expression pattern in human a human ovary section (37 years of old). Insert shows *Misr2* positive theca and granulosa cells. Scale bars: 50μm.

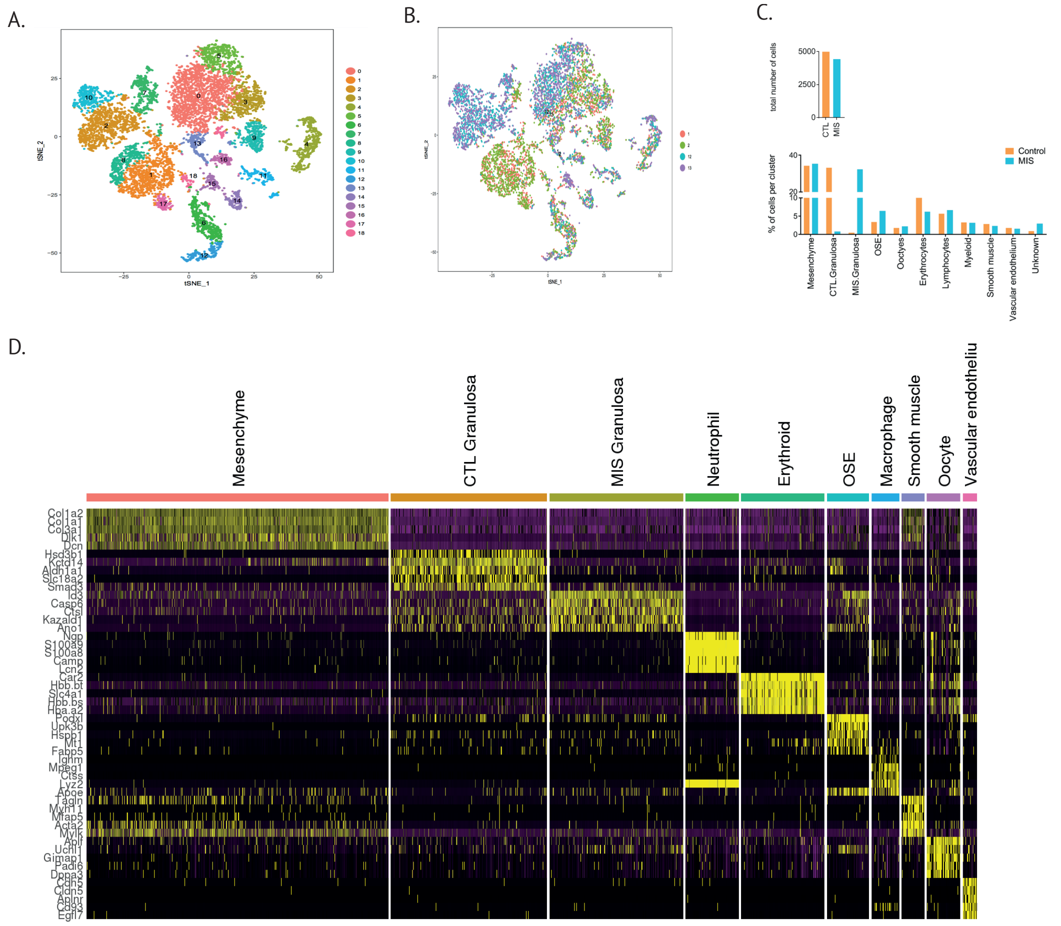

Fig. S2. ScRNAseq of ovaries from control and MIS treated mice. A. inDROP libraries were sequenced, demultiplexed, normalized, and analyzed using the Seurat package in 'R'. The processed sample of 9437 cells clustered into 18 groups. **B.** t-SNE plot of unbiased clustering of ovarian cells color-coded by treatment with two replicates for each condition, CTL (orange and green) and MIS (blue and purple). **C.** Total number of cells from CTL and MIS mouse ovaries represented in the t-SNE plots (A, B). Percentage of different cell types presented in CTL (orange) and MIS (blue) cells. **D.** Heatmap of top 10 markers per cluster ordered by logFc values.

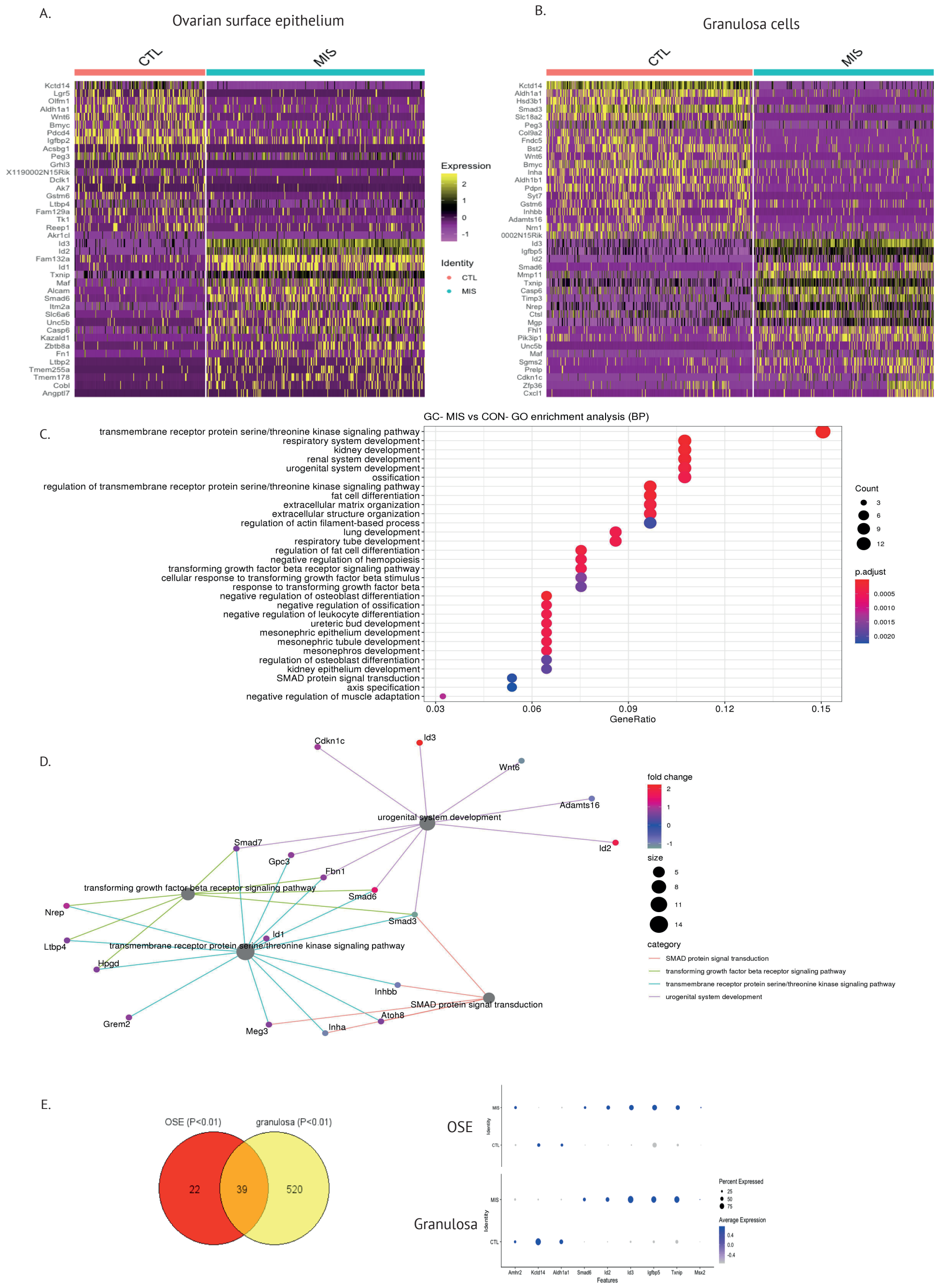

\

Fig. S3. Comparison of gene signatures associated with MIS treatment in OSE and granulosa cells. A. Heatmap of differentially expressed genes in ovarian surface epithelium genes of PND6 CTL and MIS mice ovaries. **B.** Heatmap of differentially expressed genes in PND6 CTL

and MIS mice ovaries. **C**. Pathway analysis reveals enriched pathways of differentially expressed genes (DEG) between the MIS treated granulosa cells and controls. The top 30 GO terms in which the DEG were enriched are shown. The x-axis shows the gene ratio (the percentage of total DEGs in the given GO terms). “Count”, reflected by the dot size, represents the number of genes enriched in a GO term and dot color represents the adjusted p values. **D**. Gene networks of selected pathways are shown. Node size represents the number of genes enriched in each pathway. Dot color, as shown in “fold change” color, indicates if the gene is upregulated or downregulated in MIS-treated samples. GO, Gene ontology; BP, Biological Process.

E. Venn diagram showing the number of commonly expressed genes in mouse GC and OSE (left panel). Expression levels and percentages of commonly regulated genes in granulosa cell and OSE in CTL and MIS treated ovaries (right panel).

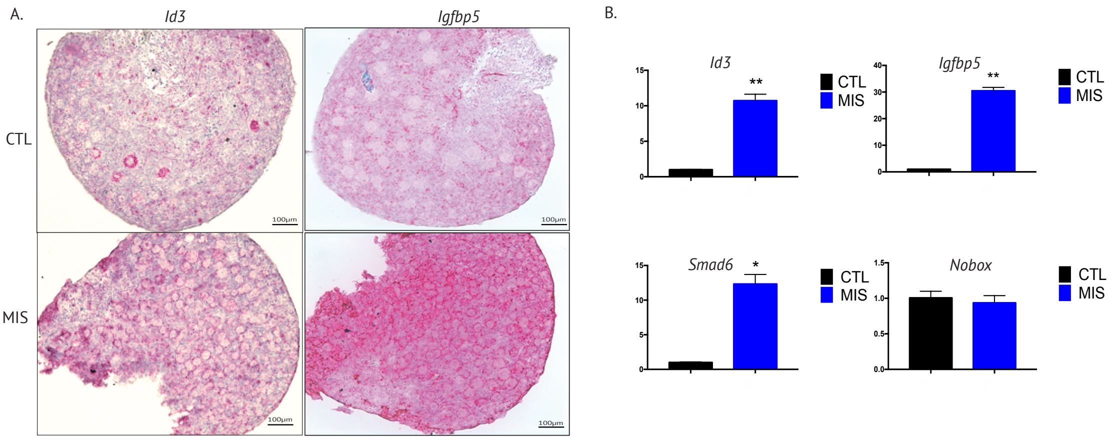

Fig.S4. ***In vitro*** **ovary culture confirms MIS directly regulates ovarian targets**

A, B. Rat ovaries were harvested on PND2 and cultured on transwell inserts in the presence of vehicle (mock purification control) or recombinant MIS (10 µg/ml) for 48 hours. **A.** *Id3, and Igfbp5* expression pattern on cultured mouse ovaries by RNAish. **B.** *Nobox (germ cell marker), Igfbp5, Smad6*, and *Id3* expression pattern by RT-qPCRs. Scale bars: 50µm. (n=3 for CTL and MIS; *$\leq$0.05; **$\leq$0.01; ***$\leq$0.001; unpaired student's t test).

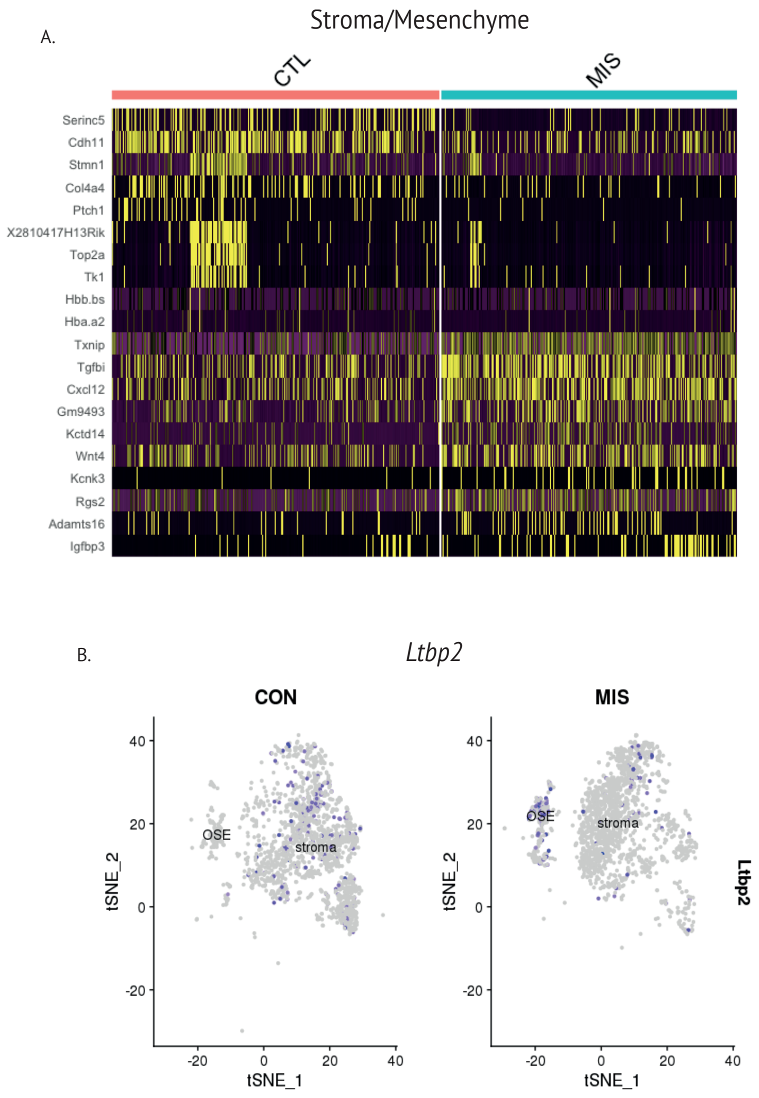

Fig.S5. Genes regulated by MIS in the ovarian stroma. A. Top 10 differentially expressed genes (by logFc) in the stroma/mesenchyme in response to MIS treatment revealed by scRNAseq in PND6 mice ovaries B. t-SNE feature plot of Ltbp2 expression based on scRNAseq in stroma/mesenchyme and ovarian surface epithelium clusters of AAV9 MIS treated mouse ovaries compared to controls.

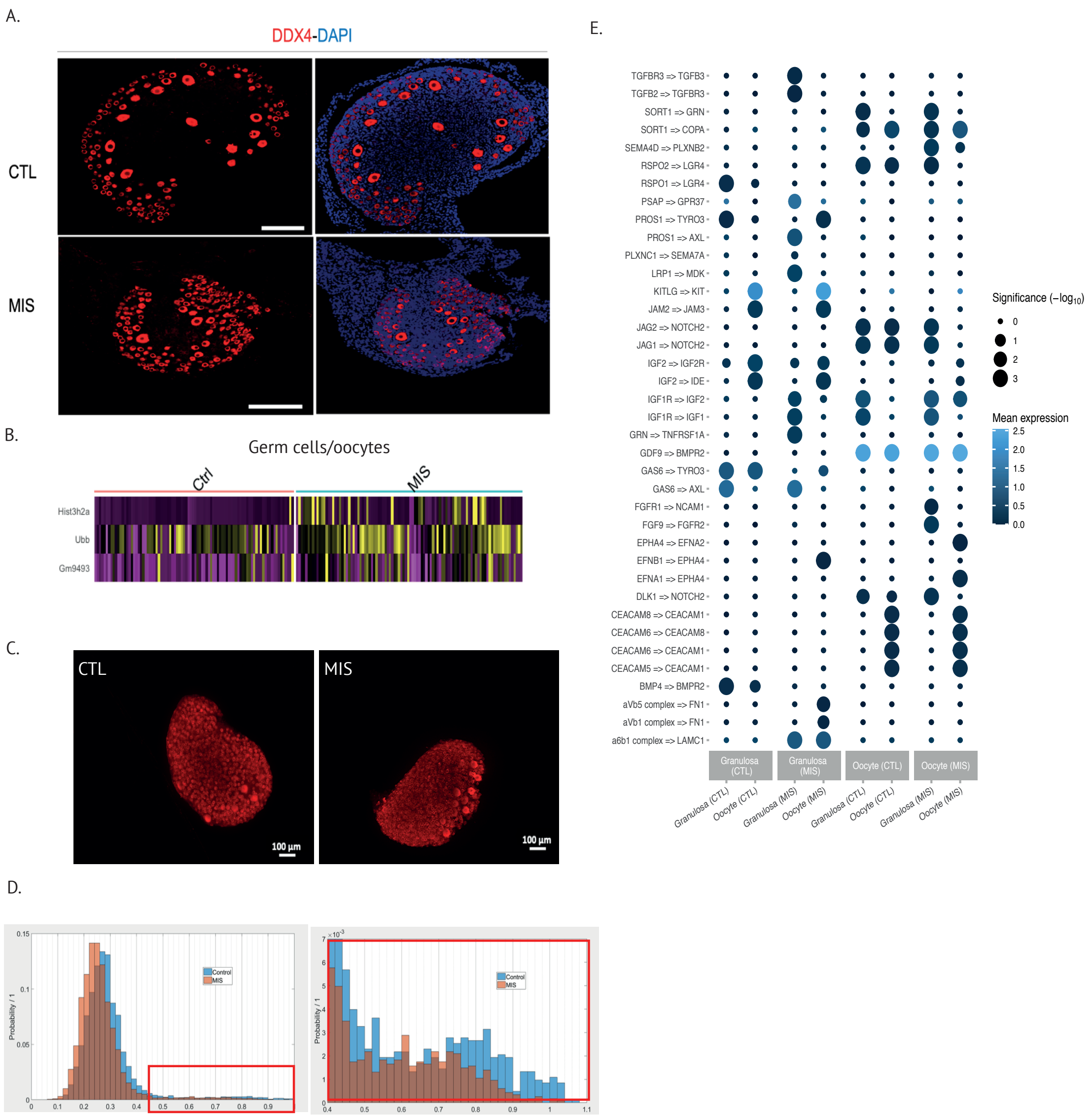

Fig.S.6 Genes regulated by MIS in germ cells. A. Immunofluorescence of DDX4 expression pattern (red) in ovarian germ cells from PND6 rat ovaries. Images are representative of three replicates Scale bars: 50µm. **B.** Heatmap of differentially expressed genes (by logFc) in germ cells in response to MIS treatment revealed by scRNAseq in PND6 mice ovaries. **C.** Whole mount DDX4 immunofluorescence of PND6 CTL and MIS treated mice ovaries. **D**. Distribution of oocytes size in CTL (blue) and MIS (orange) ovaries. **E.** CellphoneDB analysis plot indicating significantly enriched

interacting pairs ligand/receptor in oocytes and granulosa cells and their level of expression in control and MIS-treated ovaries.

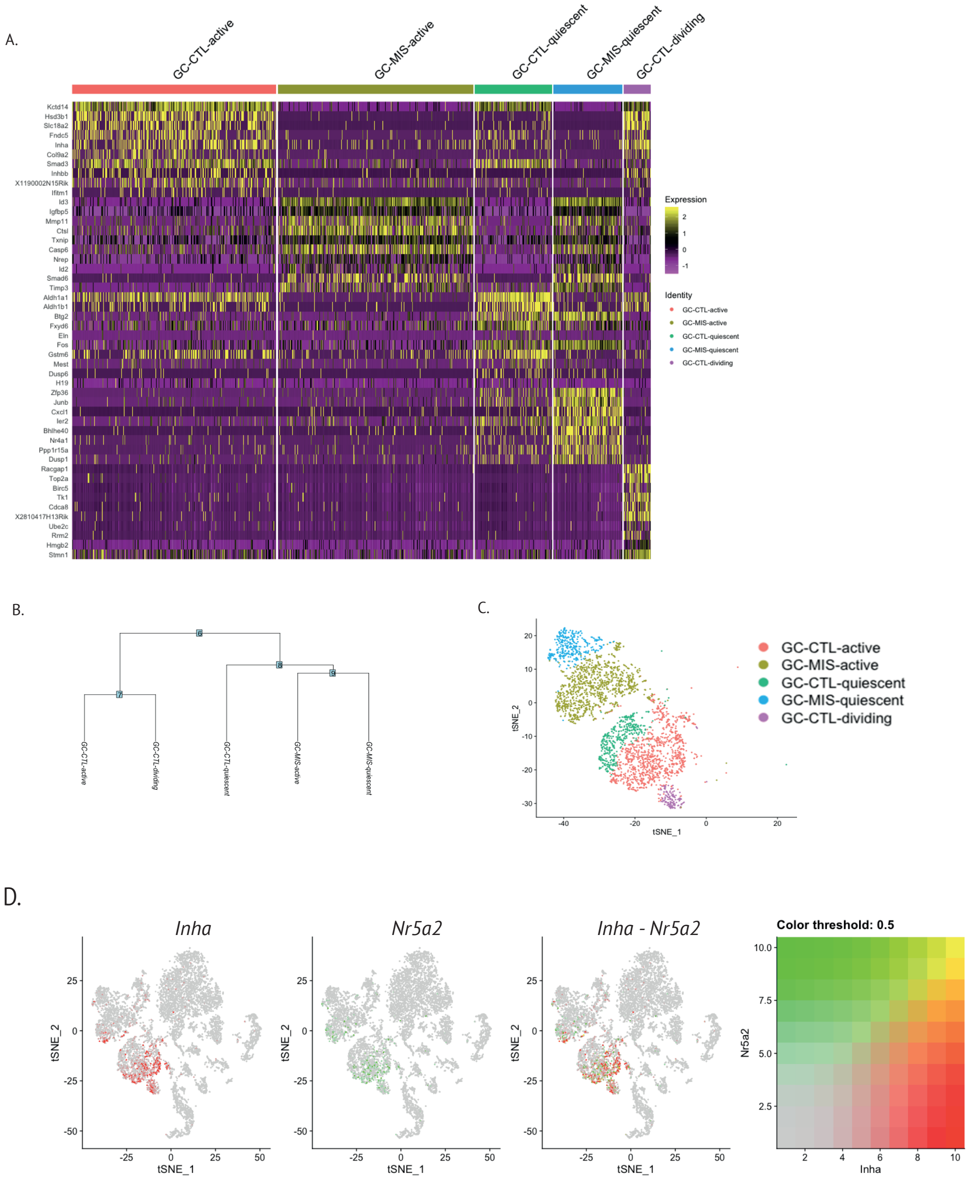

Fig.S.7 MIS modifies granulosa cell quiescence signature. A. Heatmap of differentially expressed genes in quiescent, active and dividing granulosa cells of PND6 CTL and MIS mice ovaries. **B**. Phylogenetic cluster tree showing the relatedness of all 5 granulosa cell clusters in CTL and MIS treated PND6 mouse ovaries **C.**t-SNE plot of unbiased clustering of ovarian granulosa cells color-coded by treatment and activation state with two replicates for each condition, CTL

(orange, dark green and purple) and MIS (light green and blue). **D.** Gene expression levels of granulosa activation related markers Inha (red) and Nr5a2 (green), t-SNE feature plots in PND6 CTL and MIS mouse granulosa cells.

Table S1 – Top 10 markers for clusters identification

Cluster	avg_logFC	Gene
**Mesenchyme**	2.681731241	Col1a2 (57)
	2.426715515	Col1a1 (58)
	2.231796312	Col3a1 (59)
	2.220662936	Dlk1 (60)
	2.203546419	Dcn (61)
	2.033879644	Mmp2 (62)
	1.979688906	Col6a2 (63)
	1.952594449	Mfap4 (64)
	1.90365522	Sfrp1 (65)
	1.898175634	Igfbp4 (66)
**CTL Granulosa**	2.21149334	Hsd3b1 (67)
	2.066166475	Kctd14 (68 , 69)
	1.778017951	Aldh1a1 (70)
	1.687305139	Slc18a2 (71, 72)
	1.553955185	Smad3 (73)
	1.470725208	Gstm6 (71, 74)
	1.422156259	Inha (75)
	1.396028271	Gstm2 (22, 71, 76)
	1.369783191	Bst2 (77)
	1.341566615	Aldh1b1 (78)
**MIS Granulosa**	1.84904245	Id3 (79)
	1.55901932	Casp6 (80-82)
	1.480957863	Ctsl (83, 84)
	1.470471089	Kazald1 (85)
	1.466357495	Ano1 (86)
	1.396540165	Mmp11 (87)
	1.362474146	Smad6 (82)
	1.25285887	Gatm (88)
	1.224898451	Maged2 (89)
	1.227084443	Mphosph8 (90)
**Neutrophil**	5.512040205	Ngp (82)
	5.408570381	S100a9 (91)
	4.723247555	S100a8 (91)
	4.552326401	Camp (92)
	4.492299065	Lcn2 (93)
	4.23072377	Retnlg (94)

	4.22612017	Ltf (95)
	3.636605192	Gm5483 (96)
	3.607790684	Stfa3 (97)
	3.593262497	Mmp8 (98)
**Erythroid**	3.931310846	Car2 (99)
	3.680661451	Hbb.bt
	3.444048278	Slc4a1 (100)
	3.388526687	Hbb.bs
	3.338885906	Hba.a2 (101)
	3.141003349	Hba.a1 (101)
	3.084759845	Ctse (102)
	3.05129215	Cd24a (103)
	2.777866081	Gypa (104)
	2.769264372	Alas2 (105)
**OSE**	2.484623229	Podxl (106)
	2.307910313	Upk3b (34)
	1.970740374	Hspb1 (107)
	1.856024791	Mt1 (108)
	1.759495772	Aldh1a2 (109)
	1.655022252	Krt19 (110)
	1.648674521	Bcam (111)
	1.608532073	Krt8 (110)
	1.769169838	Fabp5 (112)
	1.662323538	Fam132a
**Macrophage**	2.405539123	Ighm (113)
	2.348701901	Mpeg1 (114)
	2.235712003	Ctss (115)
	2.119377351	Plac8 (116)
	2.076980963	Laptm5 (117)
	2.053858965	Csf1r (118)
	1.784854474	Ccr2 (119)
	2.053200783	Srgn (120)
	2.209001112	Lyz2 (121)
	2.757379456	Apoe (122)
**Smooth muscle**	2.881490753	Tagln (123)
	2.418678396	Myh11 (124)
	2.22617932	Mfap5 (124)
	1.635765657	Cnn1 (125)
	3.384305646	Acta2 (126)
	1.697341618	Mcam (127)

	2.239329717	Mylk (128)
	1.710702157	Myl9 (129)
	1.487065828	Tnc (130)
	1.40318211	Pdgfrb (131)
**Oocyte**	3.637764936	Aplf (132)
	2.835278903	Uchl1 (133)
	2.763034197	Gimap1 (134)
	2.729382512	Padi6 (135)
	2.70140379	Dppa3 (136)
	2.645918984	Vmn1r65
	2.317338597	Ooep (137)
	2.075198874	Sycp3 (138)
	2.074297944	Gm13465
	2.112808315	Nlrp5 (139)
**Vascular endothelium**	2.761350871	Cdh5 (140)
	2.609404726	Cldn5 (141)
	2.548447286	Aplnr (142)
	2.487363142	Cd93 (143)
	2.433784187	Egfl7 (144)
	2.410075109	Pecam1 (145)
	2.360934838	Kdr (146)
	2.316154533	Emcn (147)
	2.264974144	Esam (148)
	2.190972032	Igfbp7 (149)

Table S2. List of RNA scope probes used to analyze the Misr2 expression pattern in rat, mouse, and human ovaries and to validate gene markers identified by the scRNAseq analysis.

Gene	Species	Accession number	Channel type	ACD
Aldh1a1	Mouse	NM_013467.3	C1	491321
Cxcl14	Mouse	NM_019568.2	C1	534881
Figla	Mouse	NM_012013.2	C2	585571
Id3	Mouse	NM_008321.2	C1	445881
Igfbp5	Mouse	NM_010518.2	C1	425731
Inhba	Mouse	NM_008380.1	C2	455871
Kcknk3	Mouse	NM_010608.2	C1	534881
Kctd14	Mouse	NM_001136235.1	C1	517811
Krt79	Mouse	NM_146063.1	C2	436201
Lgr5	Mouse	NM_010195.2	C1	312171
Misr2	Rat	NM_030998.1	C1	517791
Misr2	Mouse	NM_144547.2	C1 and C2	489821
Misr2	Human	NM_020547.2	C1	490241
Msx2	Mouse	NM_013601.2,	C1	421851
Nr5a2	Mouse	NM_030676.3	C1	543571
Ptch1	Mouse	NM_008957.2	C1	402811
Slc18a2	Mouse	NM_172523.3	C1	425331
Smad6	Mouse/Rat	NM_008542.3	C1	517781
Tmem184a	Mouse	NM_001161548.1	C1	517801

Table S3. List of qPCR primers employed to quantify mouse genes

Gene	Forward Primers Mouse	Reverse Primers Mouse
Aldh1a1	ATACTTGTCGGATTTAGGAGGCT	GGGCCTATCTTCCAAATGAACA
Cxcl12	TGCATCAGTGACGGTAAACCA	TTCTTCAGCCGTGCAACAATC
Cxcl14	GAAGATGGTTATCGTCACCACC	CGTTCCAGGCATTGTACCACT
Depdc7	ACAGAGACCTCCGGGTTTCA	TGGTGCCTTCGCTTCTTCAC
Egr1	GGAGAGGCAGGAAAGACATAAA	GCTCTGAGATCTTCCATCTGAC
Fam132a	ATTACCACGTCCAACCGTGAG	GGCCACGACACCGTTTTCT
Figla	CCGCCATCTGTAGGCTCAAG	ACACAGCCGAGTATCTGTATGTA
Fos	AATGGTGAAGACCGTGTCAG	GGATTCTCCGTTTCTCTTCCTC
Fxyd6	TCTCCGTTGGGATACTTCTCAT	CTCCGCAGCGTTTGTAGTGAT
Gapdh	GGGGCTCTCTGCTCCTCCCTG	CCAGGCGTCCGATACGGCCA
Hprt	ATGATCAGTCAACGGGGGAC	GAGAGGTCCTTTTCACCAGCA
Id3	CTGTCGGAACGTAGCCTGG	GTGGTTCATGTCGTCCAAGAG
Ifitm1	GACAGCCACCACAATCAACAT	CCCAGGCAGCAGAAGTTCAT
Igfbp3	CCAGGAAACATCAGTGAGTCC	GGATGGAACTTGGAATCGGTCA
Igfbp5	CCCTGCGACGAGAAAGCTC	GCTCTTTTCGTTGAGGCAAACC
Junb	TCACGACGACTCTTACGCAG	CCTTGAGACCCCGATAGGGA
Kcnk3	ACGGAGGCAAGGTGTTCTG	ACGACACGAAACCGATGAGC
Kctd14	CAAAGGTCATCTATGGAGCCAG	GAGAAGCCCAAAGTAGGTGCC
Kit	GCCACGTCTCAGCCATCTG	GTCGCCAGCTTCAACTATTAACT
Krt79	GTCCAGGCAGACTTTCTCCAC	ACATAGTCACTGAGCTGTAGCTG
Lgr5	CCTACTCGAAGACTTACCCAGT	GCATTGGGGTGAATGATAGCA
Ltbp2	AACAGCACCAACCACTGTATC	CCTGGCATTCTGAGGGTCAAA
Misr2	GGGGCTTTGGACACTGCTT	GTCTCGGCATCCTTGCATCTC
Msx2	CAAGAAGCCGCCCAAGGAAT	TGCTCCGTCTTCGGAATTTTC
Nobox	AAGACCCGAACCCTGTACC	CTCATGGCGTTTGTCACTGTC
Nr5a2	TGAGGAACAACTCCGGGAAAA	CAGACACTTTATCGCCACACA
P63	TACTGCCCCGACCCTTACAT	GCTGAGGAACTCGCTTGTCTG
Prelp	TGGCTCCTCCCACTTCTCC	CGTGGACAGTCAGGGAAGAC
Ptch1	AAAGAACTGCGGCAAGTTTTTG	CTTCTCCTATCTTCTGACGGGT
Rpl19	CTGAAGGTCAAAGGGAATGTG	GGACAGAGTCTTGATGATCTC
Slc18a2	CACCGTCGTAGTTCCCATCA	CTGGCAGTCTGGATTTCCGT
Smad6	GCAACCCCTACCACTTCAGC	GTGGCTTGTACTGGTCAGGAG
Stmn1	TCTGTCCCCGATTTCCCCC	AGCTGCTTCAAGACTTCCGC
Tgfbi	CAGCACGGCCCCAATGTAT	GGGACCTTTTCATATCCAGGACA

Th	GTCTCAGAGCAGGATACCAAGC	CTCTCCTCGAATACCACAGCC
Tmem184a	ATGAGGAATGCGTCAGGGTTT	AGGAACGTAGGTGGGAGTAGA
Top2a	CAACTGGAACATATACTGCTCCG	GGGTCCCTTTGTTTGTTATCAGC
Zp3	ATGGCGTCAAGCTATTTCCTC	CGTGCCAAAAAGGTCTCTACT

Table S4. List of qPCR primers employed to amplify rat genes

Gene	Forward Primers Rat	Reverse Primers Rat
Aldh1a1	GGACGAGAACTGGGTGAACA	AGGGAGCTACGACAGAAAGC
Cxcl12	GTAGACCCCTGAGGAAGGCT	TGGTGGAGTGTCTTTGTGCT
Cxcl14	TGGTCAAGAGTTTCGGTGTG	GCTCATCCTGGGAAGTAGATTG
Depdc7	CAGGCTGTCTCCTCAAGTTATC	AGGTACAACCTCTTGCTGTTT
Egr1	CGAGCACCTGACCACAGAGTC	GCCACAAAGTGTTGCCACTG
Fam132a	CTGTGACGGTAGACAAGAAGAC	CAGAGACTGGCGCTGTAAAT
Figla	AAAGAGCGTGAGCGGATAAA	TTCCTGCTCTGTGGTAGAAATG
Fos	CGACCATGATGTTCTCGGGT	TCGGCTGGGGAATGGTAGTA
Fxyd6	CCTGAGGATTGGGGGATTGG	ACTTGCACCTGCGACTTAGG
Gapdh	GGGGCTCTCTGCTCCTCCCTG	CCAGGCGTCCGATACGGCCA
Hprt	CTCATGGACTGATTATGGACAGGAC	GCAGGTCAGCAAAGAACTTATAGCC
Id3	TCAGCTTAGCCAGGTGGAAA	TGAGCTCAGCTGTCTGGATCG
Ifitm1	TCTTGTGGACGACTCCTAGAT	CTGTTCCTTGGGCATCTCTT
Igfbp3	CCGAGAGCCCATAAAGACAAG	GACATCTCTGCACATGGAGTT
Igfbp5	GGTAAAGCCAGACTCGGAAA	GGGTTCACAATGCACGAAAG
Junb	CCCGGATGTGCACGAAAATG	TGTCTGATCCCTGACCCGAA
Kcnk3	ACATGGTGCTCATCGGTTT	GTGAGGGTGATGAAGCAGTAG
Kctd14	GGAGAAGGACATTAGAGCCAAG	CCACCAAGTGAAGACGAGTATC
Kit	TTAAAGGTAACAGCAAAGAGCAAA	GTGGCCTCAACTACCTTCCC
Krt79	CTGCTGACACCTCTCAATGT	GTTCCAGGAAGCGTACCTTATC
Lgr5	CTCCCAGGTCTGGTGTGTTG	GAGGTCTAGGTAGGAGGTGAAG
Ltbp2	TTGGCTCAGACACCACAAATA	CTTCTGATGGGCGCTCTAAA
Misr2	CTGCGCTTCTCCCAGGTAAT	CTGGTACACAGCTCGCTCAG
Msx2	GCCGCCCAGACACATGA	AAGGTTCAGAGAGCTGGAGAAC
Nobox	AGTGACAGGGACCTCGATAA	CCACCAACTTGGCTATCCTT
Nr5a2	TCATGCTGCCCAAAGTGGAGA	TGGTTTTGGACAGTTCGCTT
P63	TAGCACTAGGGACTCGGAAA	GGCTACTCACACGCTTAACA
Prelp	GTCTGTGGTTATCTAGGCTGTG	AGCAAGAGAGGGAGACAGATA
Ptch1	GGAGCAGATTTCCAAGGGGA	TCTCCTATCTTCTGGCGGGT
Rpl19	CTTAGGCTACAGAAGAGGCTTG	GAGTTGGCATTGGCGATTTC
Slc18a2	ATCGCTGCAGGCTCCATCT	TGCCACTTTCGGGAACACA
Smad6	AAGCCACTGGATCTGTCCGA	GCCCTGAGGTAGGTCGTAGA
Stmn1	TCATCTGCTGAGACCCATGC	GGACCAGACAGCGGTAGAAC
Tgfbi	GTTCTATGCGACTTGCCCCT	CCGTGTTTGGAGGGATCGAA
Th	CCCTACCAAGATCAAACCTACC	CTGGATACGAGAGGCATAGTTC
Tmem184a	TGGCTACCAGAATTTCCTCATC	GGGCTGGTGAGTTCTTCTTT

Zp3	CGCCAACAGCTCCAGAAATA	CTTGTTGAAGGAACAGGCTTTG

Dataset S1 (separate file)

Cluster markers identified in PND6 ovary combined scRNAseq dataset

Dataset S2 (separate file)
Pepin D (2021) Single-cell sequencing of neonatal ovaries treated with MIS/AMH. Available at: osf.io/3sbtp.

References

1. Pepin D, *et al.* (2013) An albumin leader sequence coupled with a cleavage site modification enhances the yield of recombinant C-terminal Mullerian Inhibiting Substance. *Technology* 1(1):63-71.
2. Kano M, *et al.* (2017) AMH/MIS as a contraceptive that protects the ovarian reserve during chemotherapy. *Proceedings of the National Academy of Sciences of the United States of America* 114(9):E1688-e1697.
3. Kezele P & Skinner MK (2003) Regulation of ovarian primordial follicle assembly and development by estrogen and progesterone: endocrine model of follicle assembly. *Endocrinology* 144(8):3329-3337.
4. Trombly DJ, Woodruff TK, & Mayo KE (2009) Suppression of Notch signaling in the neonatal mouse ovary decreases primordial follicle formation. *Endocrinology* 150(2):1014-1024.
5. Rinaldi VD, Bloom JC, & Schimenti JC (2018) Whole Mount Immunofluorescence and Follicle Quantification of Cultured Mouse Ovaries. *Journal of visualized experiments : JoVE* (135).
6. Saatcioglu HD, *et al.* (2019) Single-cell sequencing of neonatal uterus reveals an Misr2+ endometrial progenitor indispensable for fertility. *eLife* 8.
7. Zhang H, Kay A, Forsyth NR, Liu KK, & El Haj AJ (2012) Gene expression of single human mesenchymal stem cell in response to fluid shear. *Journal of tissue engineering* 3(1):2041731412451988.
8. Millington-Ward S, *et al.* (2004) RNAi of COL1A1 in mesenchymal progenitor cells. *European journal of human genetics : EJHG* 12(10):864-866.
9. Zhang B, *et al.* (2018) Hypoxia induces endothelial-mesenchymal transition in pulmonary vascular remodeling. *International journal of molecular medicine* 42(1):270-278.
10. Kluth SM, *et al.* (2010) DLK-1 as a marker to distinguish unrestricted somatic stem cells and mesenchymal stromal cells in cord blood. *Stem cells and development* 19(10):1471-1483.
11. Dempsey SG, *et al.* (2020) A novel chemotactic factor derived from the extracellular matrix protein decorin recruits mesenchymal stromal cells in vitro and in vivo. *PloS one* 15(7):e0235784.
12. Bhoopathi P, *et al.* (2011) MMP-2 mediates mesenchymal stem cell tropism towards medulloblastoma tumors. *Gene therapy* 18(7):692-701.
13. Atkinson JC, Rühl M, Becker J, Ackermann R, & Schuppan D (1996) Collagen VI regulates normal and transformed mesenchymal cell proliferation in vitro. *Experimental cell research* 228(2):283-291.
14. Taylor SE, *et al.* (2016) Identification of Human Juvenile Chondrocyte-Specific Factors that Stimulate Stem Cell Growth. *Tissue engineering. Part A* 22(7-8):645-653.
15. Dufourcq P, *et al.* (2008) Secreted frizzled-related protein-1 enhances mesenchymal stem cell function in angiogenesis and contributes to neovessel maturation. *Stem cells (Dayton, Ohio)* 26(11):2991-3001.

16. Wu J, *et al.* (2017) Age-Related Insulin-Like Growth Factor Binding Protein-4 Overexpression Inhibits Osteogenic Differentiation of Rat Mesenchymal Stem Cells. *Cellular physiology and biochemistry : international journal of experimental cellular physiology, biochemistry, and pharmacology* 42(2):640-650.
17. Kakuta H, Iguchi T, & Sato T (2018) The Involvement of Granulosa Cells in the Regulation by Gonadotropins of Cyp17a1 in Theca Cells. *In vivo (Athens, Greece)* 32(6):1387-1401.
18. Kitasaka H, *et al.* (2018) Inductions of granulosa cell luteinization and cumulus expansion are dependent on the fibronectin-integrin pathway during ovulation process in mice. *PloS one* 13(2):e0192458.
19. Duffy DM, Ko C, Jo M, Brannstrom M, & Curry TE (2019) Ovulation: Parallels With Inflammatory Processes. *Endocrine reviews* 40(2):369-416.
20. Liew SH, Sarraj MA, Drummond AE, & Findlay JK (2011) Estrogen-dependent gene expression in the mouse ovary. *PloS one* 6(2):e14672.
21. Wang JJ, *et al.* (2020) Single-cell transcriptome landscape of ovarian cells during primordial follicle assembly in mice. *PLoS biology* 18(12):e3001025.
22. Kawai T, Richards JS, & Shimada M (2018) The Cell Type-Specific Expression of Lhcgr in Mouse Ovarian Cells: Evidence for a DNA-Demethylation-Dependent Mechanism. *Endocrinology* 159(5):2062-2074.
23. Gallardo TD, *et al.* (2007) Genomewide discovery and classification of candidate ovarian fertility genes in the mouse. *Genetics* 177(1):179-194.
24. Granados-Aparici S, *et al.* (2019) SMAD3 directly regulates cell cycle genes to maintain arrest in granulosa cells of mouse primordial follicles. *Scientific reports* 9(1):6513.
25. Rolland AD, Lehmann KP, Johnson KJ, Gaido KW, & Koopman P (2011) Uncovering gene regulatory networks during mouse fetal germ cell development. *Biology of reproduction* 84(4):790-800.
26. Myers M, Mansouri-Attia N, James R, Peng J, & Pangas SA (2013) GDF9 modulates the reproductive and tumor phenotype of female inha-null mice. *Biology of reproduction* 88(4):86.
27. Stapp AD, Gómez BI, Gifford CA, Hallford DM, & Hernandez Gifford JA (2014) Canonical WNT signaling inhibits follicle stimulating hormone mediated steroidogenesis in primary cultures of rat granulosa cells. *PloS one* 9(1):e86432.
28. Lim J & Luderer U (2011) Oxidative damage increases and antioxidant gene expression decreases with aging in the mouse ovary. *Biology of reproduction* 84(4):775-782.
29. Zhao ZH, *et al.* (2020) Single-cell RNA sequencing reveals regulation of fetal ovary development in the monkey (Macaca fascicularis). *Cell discovery* 6(1):97.
30. Salilew-Wondim D, *et al.* (2015) Polycystic ovarian syndrome is accompanied by repression of gene signatures associated with biosynthesis and metabolism of steroids, cholesterol and lipids. *Journal of ovarian research* 8:24.
31. Sèdes L, *et al.* (2013) Anti-Müllerian hormone recruits BMPR-IA in immature granulosa cells. *PloS one* 8(11):e81551.
32. Zhu G, *et al.* (2019) Transcriptomic Diversification of Granulosa Cells during Follicular Development in Chicken. *Scientific reports* 9(1):5462.

33. Hatzirodos N, *et al.* (2014) Transcriptome profiling of granulosa cells from bovine ovarian follicles during atresia. *BMC genomics* 15:40.
34. Khan A, *et al.* (2020) Evaluation of heat stress effects on cellular and transcriptional adaptation of bovine granulosa cells. *Journal of animal science and biotechnology* 11:25.
35. Liu L, *et al.* (2018) Whole-transcriptome analysis of atrophic ovaries in broody chickens reveals regulatory pathways associated with proliferation and apoptosis. *Scientific reports* 8(1):7231.
36. Sriraman V, Sinha M, & Richards JS (2010) Progesterone receptor-induced gene expression in primary mouse granulosa cell cultures. *Biology of reproduction* 82(2):402-412.
37. Binder AK, *et al.* (2013) The absence of ER-β results in altered gene expression in ovarian granulosa cells isolated from in vivo preovulatory follicles. *Endocrinology* 154(6):2174-2187.
38. Peri LE, *et al.* (2015) A novel class of interstitial cells in the mouse and monkey female reproductive tracts. *Biology of reproduction* 92(4):102.
39. Lv X, *et al.* (2019) Timely expression and activation of YAP1 in granulosa cells is essential for ovarian follicle development. *FASEB journal : official publication of the Federation of American Societies for Experimental Biology* 33(9):10049-10064.
40. Hatzirodos N, *et al.* (2014) Transcriptome profiling of granulosa cells of bovine ovarian follicles during growth from small to large antral sizes. *BMC genomics* 15:24.
41. Fan X, *et al.* (2019) Single-cell reconstruction of follicular remodeling in the human adult ovary. *Nature communications* 10(1):3164.
42. Sroussi HY, Lu Y, Zhang QL, Villines D, & Marucha PT (2010) S100A8 and S100A9 inhibit neutrophil oxidative metabolism in-vitro: involvement of adenosine metabolites. *Free radical research* 44(4):389-396.
43. Bloemen PG, *et al.* (1997) Increased cAMP levels in stimulated neutrophils inhibit their adhesion to human bronchial epithelial cells. *The American journal of physiology* 272(4 Pt 1):L580-587.
44. Schroll A, *et al.* (2012) Lipocalin-2 ameliorates granulocyte functionality. *European journal of immunology* 42(12):3346-3357.
45. Paris AJ, *et al.* (2016) Neutrophils promote alveolar epithelial regeneration by enhancing type II pneumocyte proliferation in a model of acid-induced acute lung injury. *American journal of physiology. Lung cellular and molecular physiology* 311(6):L1062-l1075.
46. Zhao X, *et al.* (2018) Beneficial Role of Neutrophils Through Function of Lactoferrin After Intracerebral Hemorrhage. *Stroke* 49(5):1241-1247.
47. Bilodeau M, *et al.* (2009) Analysis of blood stem cell activity and cystatin gene expression in a mouse model presenting a chromosomal deletion encompassing Csta and Stfa2l1. *PloS one* 4(10):e7500.
48. Patra D, Kim J, Zhang Q, Tycksen E, & Sandell LJ (2020) Site-1 protease ablation in the osterix-lineage in mice results in bone marrow neutrophilia and hematopoietic stem cell alterations. *Biology open* 9(6).

49. Lin M, *et al.* (2008) Matrix metalloproteinase-8 facilitates neutrophil migration through the corneal stromal matrix by collagen degradation and production of the chemotactic peptide Pro-Gly-Pro. *The American journal of pathology* 173(1):144-153.
50. Song SH, Kim A, Dale R, & Dean A (2012) Ldb1 regulates carbonic anhydrase 1 during erythroid differentiation. *Biochimica et biophysica acta* 1819(8):885-891.
51. McManus K, Pongoski J, Coghlan G, & Zelinski T (2000) Amino acid substitutions in human erythroid protein band 3 account for the low-incidence antigens NFLD and BOW. *Transfusion* 40(3):325-329.
52. Capellera-Garcia S, *et al.* (2016) Defining the Minimal Factors Required for Erythropoiesis through Direct Lineage Conversion. *Cell reports* 15(11):2550-2562.
53. Machherndl-Spandl S, *et al.* (2013) Molecular pathways of early CD105-positive erythroid cells as compared with CD34-positive common precursor cells by flow cytometric cell-sorting and gene expression profiling. *Blood cancer journal* 3(1):e100.
54. Hattangadi SM, Wong P, Zhang L, Flygare J, & Lodish HF (2011) From stem cell to red cell: regulation of erythropoiesis at multiple levels by multiple proteins, RNAs, and chromatin modifications. *Blood* 118(24):6258-6268.
55. Herkt SC, *et al.* (2018) Protein arginine methyltransferase 6 controls erythroid gene expression and differentiation of human CD34(+) progenitor cells. *Haematologica* 103(1):18-29.
56. Kaneko K, *et al.* (2014) Identification of a novel erythroid-specific enhancer for the ALAS2 gene and its loss-of-function mutation which is associated with congenital sideroblastic anemia. *Haematologica* 99(2):252-261.
57. Mullany LK, *et al.* (2011) Molecular and functional characteristics of ovarian surface epithelial cells transformed by KrasG12D and loss of Pten in a mouse model in vivo. *Oncogene* 30(32):3522-3536.
58. Niu W & Spradling AC (2020) Two distinct pathways of pregranulosa cell differentiation support follicle formation in the mouse ovary. *Proceedings of the National Academy of Sciences of the United States of America* 117(33):20015-20026.
59. Emmanuel C, *et al.* (2011) Comparison of expression profiles in ovarian epithelium in vivo and ovarian cancer identifies novel candidate genes involved in disease pathogenesis. *PloS one* 6(3):e17617.
60. Knapinska AM & Fields GB (2019) The Expanding Role of MT1-MMP in Cancer Progression. *Pharmaceuticals (Basel, Switzerland)* 12(2).
61. Williams SJ, Cvetkovic D, & Hamilton TC (2009) Vitamin A metabolism is impaired in human ovarian cancer. *Gynecologic oncology* 112(3):637-645.
62. Wang J, Wang D, Chu K, Li W, & Zeng YA (2019) Procr-expressing progenitor cells are responsible for murine ovulatory rupture repair of ovarian surface epithelium. *Nature communications* 10(1):4966.
63. Schön M, *et al.* (2000) Basal-cell adhesion molecule (B-CAM) is induced in epithelial skin tumors and inflammatory epidermis, and is expressed at cell-cell and cell-substrate contact sites. *The Journal of investigative dermatology* 115(6):1047-1053.

64. Ohata T, *et al.* (2017) Fatty acid-binding protein 5 function in hepatocellular carcinoma through induction of epithelial-mesenchymal transition. *Cancer medicine* 6(5):1049-1061.
65. Fuchs T, *et al.* (2018) Expression of combinatorial immunoglobulins in macrophages in the tumor microenvironment. *PloS one* 13(9):e0204108.
66. Benard EL, *et al.* (2015) Macrophage-expressed perforins mpeg1 and mpeg1.2 have an anti-bacterial function in zebrafish. *Journal of innate immunity* 7(2):136-152.
67. Hughes CS, *et al.* (2016) Extracellular cathepsin S and intracellular caspase 1 activation are surrogate biomarkers of particulate-induced lysosomal disruption in macrophages. *Particle and fibre toxicology* 13:19.
68. Domingo-Gonzalez R, *et al.* (2020) Diverse homeostatic and immunomodulatory roles of immune cells in the developing mouse lung at single cell resolution. *eLife* 9.
69. Glowacka WK, Alberts P, Ouchida R, Wang JY, & Rotin D (2012) LAPTM5 protein is a positive regulator of proinflammatory signaling pathways in macrophages. *The Journal of biological chemistry* 287(33):27691-27702.
70. Sehgal A, *et al.* (2018) The role of CSF1R-dependent macrophages in control of the intestinal stem-cell niche. *Nature communications* 9(1):1272.
71. Boniakowski AE, *et al.* (2018) Murine macrophage chemokine receptor CCR2 plays a crucial role in macrophage recruitment and regulated inflammation in wound healing. *European journal of immunology* 48(9):1445-1455.
72. Zernichow L, *et al.* (2006) Serglycin is the major secreted proteoglycan in macrophages and has a role in the regulation of macrophage tumor necrosis factor-alpha secretion in response to lipopolysaccharide. *The Journal of biological chemistry* 281(37):26792-26801.
73. Murray PJ & Wynn TA (2011) Obstacles and opportunities for understanding macrophage polarization. *Journal of leukocyte biology* 89(4):557-563.
74. Tedla N, Glaros EN, Brunk UT, Jessup W, & Garner B (2004) Heterogeneous expression of apolipoprotein-E by human macrophages. *Immunology* 113(3):338-347.
75. Tawfik O, *et al.* (2014) Transgelin, a Novel Marker of Smooth Muscle Differentiation, Effectively Distinguishes Endometrial Stromal Tumors from Uterine Smooth Muscle Tumors. *International journal of gynecological obstetrical and reproductive medicine research* 1(1):26-31.
76. Barbier M, *et al.* (2014) MFAP5 loss-of-function mutations underscore the involvement of matrix alteration in the pathogenesis of familial thoracic aortic aneurysms and dissections. *American journal of human genetics* 95(6):736-743.
77. Liu R & Jin JP (2016) Calponin isoforms CNN1, CNN2 and CNN3: Regulators for actin cytoskeleton functions in smooth muscle and non-muscle cells. *Gene* 585(1):143-153.
78. Yuan SM (2015) α-Smooth Muscle Actin and ACTA2 Gene Expressions in Vasculopathies. *Brazilian journal of cardiovascular surgery* 30(6):644-649.
79. Espagnolle N, *et al.* (2014) CD146 expression on mesenchymal stem cells is associated with their vascular smooth muscle commitment. *Journal of cellular and molecular medicine* 18(1):104-114.

80. Han YJ, Ma SF, Wade MS, Flores C, & Garcia JG (2012) An intronic MYLK variant associated with inflammatory lung disease regulates promoter activity of the smooth muscle myosin light chain kinase isoform. *Journal of molecular medicine (Berlin, Germany)* 90(3):299-308.
81. Shehadeh LA, Webster KA, Hare JM, & Vazquez-Padron RI (2011) Dynamic regulation of vascular myosin light chain (MYL9) with injury and aging. *PloS one* 6(10):e25855.
82. Ando K*, et al.* (2011) Tenascin C may regulate the recruitment of smooth muscle cells during coronary artery development. *Differentiation; research in biological diversity* 81(5):299-306.
83. Wan W*, et al.* (2019) PDGFR-β modulates vascular smooth muscle cell phenotype via IRF-9/SIRT-1/NF-κB pathway in subarachnoid hemorrhage rats. *Journal of cerebral blood flow and metabolism : official journal of the International Society of Cerebral Blood Flow and Metabolism* 39(7):1369-1380.
84. Varghese PC*, et al.* (2021) Histone chaperone APLF level dictates the implantation of mouse embryos. *J Cell Sci* 134(1).
85. Mtango NR, Sutovsky M, Vandevoort CA, Latham KE, & Sutovsky P (2012) Essential role of ubiquitin C-terminal hydrolases UCHL1 and UCHL3 in mammalian oocyte maturation. *Journal of cellular physiology* 227(5):2022-2029.
86. de los Santos MJ*, et al.* (2012) Hormonal and molecular characterization of follicular fluid, cumulus cells and oocytes from pre-ovulatory follicles in stimulated and unstimulated cycles. *Human reproduction (Oxford, England)* 27(6):1596-1605.
87. Bebbere D*, et al.* (2020) Subcortical maternal complex (SCMC) expression during folliculogenesis is affected by oocyte donor age in sheep. *Journal of assisted reproduction and genetics* 37(9):2259-2271.
88. Vendrell-Flotats M*, et al.* (2020) In Vitro Maturation with Leukemia Inhibitory Factor Prior to the Vitrification of Bovine Oocytes Improves Their Embryo Developmental Potential and Gene Expression in Oocytes and Embryos. *International journal of molecular sciences* 21(19).
89. He DJ*, et al.* (2018) Maternal gene Ooep may participate in homologous recombination-mediated DNA double-strand break repair in mouse oocytes. *Zoological research* 39(6):387-395.
90. Wang Z, Liu CY, Zhao Y, & Dean J (2020) FIGLA, LHX8 and SOHLH1 transcription factor networks regulate mouse oocyte growth and differentiation. *Nucleic acids research* 48(7):3525-3541.
91. Fernandes R*, et al.* (2012) NLRP5 mediates mitochondrial function in mouse oocytes and embryos. *Biology of reproduction* 86(5):138, 131-110.
92. Sauteur L*, et al.* (2014) Cdh5/VE-cadherin promotes endothelial cell interface elongation via cortical actin polymerization during angiogenic sprouting. *Cell reports* 9(2):504-513.
93. Jang AS*, et al.* (2011) Endothelial dysfunction and claudin 5 regulation during acrolein-induced lung injury. *American journal of respiratory cell and molecular biology* 44(4):483-490.

94. Hwangbo C, *et al.* (2017) Endothelial APLNR regulates tissue fatty acid uptake and is essential for apelin's glucose-lowering effects. *Science translational medicine* 9(407).
95. Galvagni F, *et al.* (2016) CD93 and dystroglycan cooperation in human endothelial cell adhesion and migration adhesion and migration. *Oncotarget* 7(9):10090-10103.
96. Campagnolo L, *et al.* (2005) EGFL7 is a chemoattractant for endothelial cells and is up-regulated in angiogenesis and arterial injury. *The American journal of pathology* 167(1):275-284.
97. Privratsky JR & Newman PJ (2014) PECAM-1: regulator of endothelial junctional integrity. *Cell and tissue research* 355(3):607-619.
98. Kroll J & Waltenberger J (1997) The vascular endothelial growth factor receptor KDR activates multiple signal transduction pathways in porcine aortic endothelial cells. *The Journal of biological chemistry* 272(51):32521-32527.
99. Park-Windhol C, *et al.* (2017) Endomucin inhibits VEGF-induced endothelial cell migration, growth, and morphogenesis by modulating VEGFR2 signaling. *Scientific reports* 7(1):17138.
100. Inoue M, *et al.* (2010) Endothelial cell-selective adhesion molecule modulates atherosclerosis through plaque angiogenesis and monocyte-endothelial interaction. *Microvascular research* 80(2):179-187.
101. Tamura K, *et al.* (2009) Insulin-like growth factor binding protein-7 (IGFBP7) blocks vascular endothelial cell growth factor (VEGF)-induced angiogenesis in human vascular endothelial cells. *European journal of pharmacology* 610(1-3):61-67.
